# An allozyme polymorphism is associated with a large chromosomal inversion in the marine snail *Littorina fabalis*

**DOI:** 10.1111/eva.13427

**Published:** 2022-06-26

**Authors:** Alan Le Moan, Marina Panova, Aurélien De Jode, Olga Ortega‐Martinez, Mårten Duvetorp, Rui Faria, Roger Butlin, Kerstin Johannesson

**Affiliations:** ^1^ Tjärnö Marine Laboratory, Department of Marine Sciences University of Gothenburg Strömstad Sweden; ^2^ InBIO Laboratório Associado, CIBIO, Centro de Investigação em Biodiversidade e Recursos Genéticos Universidade do Porto Vairão Portugal; ^3^ BIOPOLIS Program in Genomics, Biodiversity and Land Planning, CIBIO Campus de Vairão Vairão Portugal; ^4^ Ecology and Evolutionary Biology, School of Biosciences University of Sheffield Sheffield UK

**Keywords:** arginine kinase, candidate gene, hybrid zones, nonsynonymous substitutions, speciation

## Abstract

Understanding the genetic targets of natural selection is one of the most challenging goals of population genetics. Some of the earliest candidate genes were identified from associations between allozyme allele frequencies and environmental variation. One such example is the clinal polymorphism in the arginine kinase (*Ak*) gene in the marine snail *Littorina fabalis*. While other enzyme loci do not show differences in allozyme frequencies among populations, the *Ak* alleles are near differential fixation across repeated wave exposure gradients in Europe. Here, we use this case to illustrate how a new sequencing toolbox can be employed to characterize the genomic architecture associated with historical candidate genes. We found that the *Ak* alleles differ by nine nonsynonymous substitutions, which perfectly explain the different migration patterns of the allozymes during electrophoresis. Moreover, by exploring the genomic context of the *Ak* gene, we found that the three main *Ak* alleles are located on different arrangements of a putative chromosomal inversion that reaches near fixation at the opposing ends of two transects covering a wave exposure gradient. This shows *Ak* is part of a large (3/4 of the chromosome) genomic block of differentiation, in which *Ak* is unlikely to be the only target of divergent selection. Nevertheless, the nonsynonymous substitutions among *Ak* alleles and the complete association of one allele with one inversion arrangement suggest that the *Ak* gene is a strong candidate to contribute to the adaptive significance of the inversion.

## 1 INTRODUCTION

For many decades, a central question in population genetics has been to understand how divergent natural selection promoting local adaptation interacts with other microevolutionary processes to shape genetic differences among populations. Early suggestions of candidate genes involved in local adaptation were described at allozyme loci using electrophoresis in the 1970–1990ies (Hedgecock, 1986; Hilbish et al., 1982; Merçot et al., 1994; Oakeshott et al., 1982). However, in most cases, it has remained unclear whether the allozyme polymorphisms are caused by divergent selection acting directly on the candidate locus, by hitchhiking effects or by other types of selection (Bierne et al., 2011). Similar issues apply to outlier loci detected more recently using genome scans as candidates for a role in local adaptation (Ravinet et al., 2017). For example, loci located in a low‐recombining region are likely to be detected as outliers due to background selection reducing diversity at linked sites (Burri, 2017; Cruickshank & Hahn, 2014). Until recently, the genomic architecture of adaptation remained unknown for most groups of organisms, with the notable exception of some species of flies where early studies located outlier loci to large inversions (Day et al., 1982; Oakeshott et al., 1982). Thus, exploring the genomic context of candidate genes, including allozyme loci, is necessary for critical tests of their role in local adaptation (Nunez et al., 2020).

Chromosomal rearrangements, and inversions, in particular, are known to reduce effective recombination among alleles at different loci involved in local adaption, facilitating local adaptation under gene flow (Jones et al., 2012; Kirkpatrick & Barton, 2006; Mérot et al., 2020). Using new sequencing approaches, an increasing number of empirical examples of rearrangements involved in local adaptation have been reported (Le Moan et al., 2021; Shi et al., 2021; Tepolt et al., 2022; Whiting et al., 2021). For instance, in the marine snail, *Littorina saxatilis*, polymorphism in several chromosomal inversions underlies major genetic differences between a large ecotype adapted to crab predation and a small ecotype adapted to wave action (Faria et al., 2019; Morales et al., 2019). Furthermore, multiple SNPs within these inversions show sharp allele frequency clines associated with fine‐scale (~150 m) switches from crab‐rich to wave‐exposed parts of rocky shores (Westram et al., 2018, 2021). Some of these inversions have been shown to carry QTLs coding for adaptive phenotypic differences between the two ecotypes (Koch et al., 2021). Here, a key question is whether a candidate gene found inside an inversion contributes more than marginally to the adaptive significance of the inversion, or is rather a hitchhiker showing increased divergence due to selection targeting other loci inside the inversion.

A related species, the flat periwinkle, *Littorina fabalis*, lives in the intertidal macroalgae (fucoid) zone on the European coast of the Atlantic. In many places, a large and a dwarf ecotype occur along the same shores but occupy different microenvironments across wave exposure gradients (Reimchen, 1981). The large ecotype is about 1.5 times larger than the dwarf ecotype and is generally found in more wave‐exposed parts of the shore than the dwarf ecotype. Early population genetic work by Tatarenkov and Johannesson (1994) found no, or very weak, genetic differences at three allozyme loci between Swedish populations of the two ecotypes, which strongly contrasted with the arginine kinase locus (*Ak* hereafter). Three major alleles, *Ak* ^80^, *Ak* ^100^, and *Ak* ^120^ were identified at this locus with different migration distances in electrophoresis gels likely due to different contents in charged amino acids. The *Ak* ^120^ was nearly fixed in the dwarf ecotype and at minor frequency in the large ecotype. In areas of intermediate wave exposure where the two ecotypes overlap in distribution, a strong association between the *Ak* alleles and adult snail size was found (Tatarenkov & Johannesson, 1998). The pronounced *Ak* polymorphism over the two microenvironments was replicated in samples from Britain and France where similar ecotypes have been described, but not in Spain (Tatarenkov & Johannesson, 1999). A later study also failed to detect genetic differences between the two ecotypes in samples from Sweden, Norway, and Shetland using four microsatellite loci (Kemppainen et al., 2009). However, in a recent study, Galindo et al. (2021) found ecotype differentiation based on a high number of AFLP outliers, while nonoutliers instead showed population structure compatible with geography.

The lack of differentiation in many loci suggests ongoing gene flow between the two ecotypes over large parts of the genome. In contrast, the sharp allele frequency differences at the *Ak* locus and other outliers between populations facing different wave exposures suggest that this allozyme is a good candidate for being involved in local adaptation. Although other outliers are anonymous, AK is known to catalyze the reversible phosphate transfer from phosphoarginine to ADP, where phosphoarginine acts as an energy store and an ATP buffer in invertebrate cells with high and variable rates of energy turnover (Ellington, 2001; Hochachka & Somero, 2014; Uda et al., 2006). In *L. fabalis*, the AK enzymatic activity was significantly higher in muscle tissue from snails' foot than in hepatopancreas (Panova et al., unpublished results). Hence, the kinetic properties of these allozymes could be under differential selection to provide proper adherence to snails living under variable wave action. However, earlier studies also found strong linkage disequilibrium (LD) between *Ak* alleles and snail size in transect centers (Tatarenkov & Johannesson, 1999), and between *Ak* and an anonymous DNA locus (a RAPD marker; Johannesson & Mikhailova, 2004), suggesting that the *Ak* gene could be located in a low‐recombining region, such as a chromosomal inversion. This would also be consistent with the many AFLP outliers observed by Galindo et al. (2021). In addition, early sequencing of one *Ak* intron confirmed the presence of divergent alleles distinguishing the two ecotypes (Kemppainen et al., 2011). However, some individuals carried more than two alleles, suggesting that a duplication could be associated with the *Ak* polymorphism.

Here, we aimed to explore in greater detail the genomic background surrounding the arginine kinase locus and its consequences for divergence between the large and the dwarf *L. fabalis* ecotypes. First, we characterized the whole *Ak* coding region by sequencing transcripts and found a similar allelic variation to the allozyme study. Notably, we found that the alleles differ by a large number of nonsynonymous substitutions. Then, using whole‐genome sequencing (WGS), we found that the *Ak* gene was located within a large LD block. Assuming synteny with the reference genome of the close relative *L. saxatilis*, this LD‐block covers most of one chromosome, consistent with the presence of a large chromosomal inversion. We then tested whether or not the *Ak* gene stands out as an outlier within this putative inversion. Altogether, our study highlights the importance of revisiting allozyme‐based historical findings on divergent evolution using contemporary population genomics tools to gain a better understanding of the genomic architecture of adaptation.

## 2 MATERIALS AND METHODS

### 2.1 Characterization of *Ak* sequences

#### 2.1.1 Sampling

For the gene characterization, we collected *L. saxatilis* and *L. fabalis* individuals of the two ecotypes in Sweden (Tjärnö, Strömstad, N58°53′, E11°7′) and the UK (Anglesey, Wales, N53°14′, W4°35′) in 2010. Samples for RNA extraction were obtained from fresh foot tissue and stored in RNAlater® (Ambion) solution at −20°C. The remaining tissue was homogenized in Tris–EDTA–boric acid buffer (pH 8.6) for allozyme electrophoresis and stored at −80°C.

#### 2.1.2 Sequencing the whole coding DNA sequence (CDS) for *Ak* in *L. saxatilis*

The full coding sequence for the *Ak* was first obtained for *L. saxatilis* based on two partial transcripts of the gene showing high similarity to the AK protein sequences in other species. These partial transcripts were representing the 196 N‐terminal and 150 C‐terminal amino acids, from an expressed sequence tags library for *L. saxatilis* (Canbäck et al., 2012). Two primers Arke‐2F (CCAACTTCGGCAAGGAGAAT) and Arke‐4R (CCTTCTGCATGGAGATGAGC) were designed to amplify the missing middle part of the CDS. Total RNA was extracted for six individuals of *L. saxatilis* using the E.Z.N.A. Mollusc RNA kit (Omega Bio‐Tek), and single‐stranded cDNA was produced using AMV Reverse Transcriptase from Promega, following the manufacturer's oligo‐dT primer protocol. This single‐stranded cDNA was used as template for *Ak* cDNA amplification. PCR products were cloned using the TOPO TA cloning kit (Invitrogen), and eight clones per individual were Sanger‐sequenced in forward and reverse directions. The full CDS, obtained by Sanger sequencing, was aligned to the *L. saxatilis* reference genome (Westram et al., 2018) using BLAT (Kent, 2002) to retrieve the contig containing the *Ak* gene. Exon and intron boundaries were further identified with the Exonerate tool (Slater & Birney, 2005).

#### 2.1.3 Sequencing the CDS for Ak in *L. fabalis*

First, *Ak* allozyme (EC 2.7.3.3) genotypes of individual snails were identified using horizontal starch gel electrophoresis with Tris–EDTA–boric acid buffer (pH 8.6) following a modified protocol from Tatarenkov and Johannesson (1994) after running for 10 h at 20 mA and 220 V. Full CDSs were then characterized for five *L. fabalis* individuals, two homozygous for the *Ak* ^120^ allele, two homozygous for *Ak* ^100^, and one homozygous for the less common *Ak* ^80^ allele, and one *L. obtusata* homozygous for the most common allozyme allele was found in this species, *Ak* ^100‐OBT^, with the same electromorph characteristics as the *Ak* ^100^ allele in *L. fabalis*.

Based on the CDS in *L. saxatilis*, we designed the primers *Ak*‐L5R (AGCTTGGGGATCTTGATGTGCACCG), *Ak*‐HR (CGTCA CGCTT GGCGAACGACAGCTTCT), *Ak*‐L2F (CACGCCC AACTT CGGCA AGGAGAAT), and *Ak*‐HF (GTGTCCGCGT CGGCCGCT CCCATGA) to amplify the full CDS and partial UTRs of the gene in *L. fabalis* and *L. obtusata*. These primers were used together with the primers from the GeneRacer kit (Invitrogen) that amplifies the UTR's to obtain full CDS in *L. fabalis* and *L. obtusata*. RNA extractions and reverse transcription were implemented as above. We performed PCR using Phusion High Fidelity DNA polymerase (Thermo Scientific) and the Zero Blunt TOPO PCR Cloning kit (Invitrogen) according to GeneRacer kit protocols. Eight clones per PCR product were Sanger‐sequenced at Macrogen Inc. Forward and reverse sequences were manually checked and assembled using Geneious software from Biomatters. Nucleotide variants present only in a single clone (out of eight) were considered to be PCR artifacts and excluded; variants supported by two or more clones were considered as real. In addition, a partial CDS (957 nt of total 1053 nt) was characterized in 16 *L. fabalis* individuals, homozygous for *Ak* ^100^ or *Ak* ^120^ allozyme alleles from Sweden and Wales (four per country × genotype). For these partial CDSs, RNA extractions and reverse transcription were performed as above and they were amplified using primers *Ak*‐2F and *Ak*‐5R. PCR, cloning, and Sanger sequencing of eight clones per individual were done as above.

#### 2.1.4 Phylogeny of the *Ak* allele and protein structure

A tree for the partial amino acid sequences obtained from the CDS was made using a neighbor‐joining algorithm on the Jukes–Cantor distance (Jukes & Cantor, 1969) with bootstrap resampling in Geneious (Biomatters Ltd) and rooted with the *Ak* sequence for the gastropod *Conus novaehollandiae* (accession number ADK73590). To test whether the electrophoretic separation of the allozyme alleles matched the expectations from the differences found in protein structure of the CDS parts, we calculated the isoelectric point using Geneious for translated nucleotide sequences. The isoelectric point shows the relative charge of a protein and indicates how proteins of similar 3D structure separate in a gel during electrophoresis. Finally, we used the arginine kinase 3D structure derived from crystalline protein of *Limulus polyphemus* (Protein Data Bank code 1 M15, with an amino acid sequence similarity of 57% to AK120) to guide modeling of a tentative 3D structure of AK in *L. fabalis* based on the amino acid sequence of the AK120 allozyme using the SWISS‐MODEL workspace (Arnold et al., 2006). From this model, we inferred the positions of amino acid differences caused by nonsynonymous mutations between allozyme alleles.

### 2.2 Whole‐genome sequencing (WGS)

#### 2.2.1 Sampling

For the WGS analyses, 295 snails were collected in April 2018 from transects covering two wave exposure gradients on the island of Lökholmen, Sweden (N58°88′, E11°11′). We collected 128 and 167 snails from the southern and northern transects, respectively. The precise position of each snail in three‐dimensional space along the shore was recorded using a Total Station (Trimble M3; error typically <1 cm). The three‐dimensional position was then transformed into a one‐dimensional path for the downstream cline analyses following the procedure developed in Westram et al. (2021). A picture of each snail with the aperture up was taken using a camera attached to a stereomicroscope. The largest diameter irrespective of the orientation of each snail was obtained from the photograph and used as an estimate of snail size. Finally, the snails were dissected and sexed, and muscle tissue was preserved in ethanol for DNA extractions.

#### 2.2.2 DNA extraction and library preparation

DNA was extracted following the protocol by Panova et al. (2016) with cetyltrimethylammonium bromide buffer and purified with the Genomic DNA Clean & Concentrator‐5 kit (Zymo Research) using the manufacturer's protocol. The DNA concentration was measured on a Q‐bit and its purity on nanodrop. Extracted samples of DNA were shipped to SciLife, Sweden, where Nextera short‐read library preparations were conducted with 300–350 bp insert size. Individual snails were then paired‐end (150 bp)‐sequenced on an Illumina NovaSeq S6000 targeting 5× coverage, except for four individuals from each end of each transect, which were sequenced targeting 15× coverage.

#### 2.2.3 Bioinformatics pipeline

For each individual, reads were filtered and mapped to the *L. saxatilis* reference genome (Westram et al., 2018) using bwa‐mem with default parameters (Li & Durbin, 2009). An average of 98% of the reads were mapped across *L. fabalis* individuals. SNP calling was performed for contigs placed on the *L. saxatilis* genetic map (Westram et al., 2018) using GATK v4.11 (McKenna et al., 2010) following best practice guidelines, including base‐quality score recalibration and variant‐quality score recalibration (DePristo et al., 2011). Only SNPs from linkage group 3 (LG3), which carries the contig where the *Ak* gene is located, were kept to explore the genomic context of our candidate gene. Further filtering steps were carried out in vcftools (Danecek et al., 2011) to remove low‐frequency variants; that is, only alleles with a frequency above 1% and positions sequenced in at least 90% of all the individuals were kept. Markers with overall observed heterozygosity above 0.6 or an average sequencing depth above 10× were discarded to remove potential paralogous sequences. We obtained 58,246 SNPs on LG3 and 9905 after pruning for close physical linkage (1 SNP per 1 kb based on LD estimates in *L. saxatilis*; Westram et al., 2018) for the downstream analyses.

#### 2.2.4 WGS statistical analyses

##### 2.2.4.1 Population structure

Using the dataset pruned for physical linkage, genetic variation among individuals was visualized with PCAs using the R package adegenet (Jombart & Ahmed, 2011). The clustering of the samples was then explored using a discriminant analyses of principal component (DAPC) approach in adegenet (Jombart et al., 2010), with the best number of clusters determined by the find.clusters function based on AIC. Snails were then assigned to each cluster to characterize the mean observed heterozygosity across all SNPs in each cluster. Weir and Cockerham's *F*_ST_ was calculated for each SNP between snails localized at each end of the transect (i.e., at 0–25 m and at 150–175 m) and between the most differentiated clusters in the DAPC using vcftools (Danecek et al., 2011). Pairwise LD was then calculated between SNPs with a minor allele frequency of >10% in total, using the pruned set using the R package LDheatmap (Shin et al., 2006). We then repeated these analyses using SNPs from contig 265 (~71 kb), containing the *Ak* locus (70 SNPs in total).

##### 2.2.4.2 Cline fitting analyses

The analysis of population structure along LG3 revealed three clusters, while the structure of the *Ak* contig revealed six clusters of individuals (see below). These clustering patterns are consistent with a polymorphic chromosomal rearrangement and with the genotypic combinations of the three *Ak* alleles already described in allozyme studies (Tatarenkov & Johannesson, 1994, 1999; and see Results) and so were used to infer the variation in arrangement or allele frequency across transects. Cline fitting was performed using the maximum‐likelihood estimation as implemented in the R package bbmle with the function mle2 (Bolker & Team, 2010) for the two putative arrangements, the three alleles of the *Ak* locus, and each of individual SNPs with a frequency difference >10% in each transect. More precisely, we compared three models using AIC, following Westram et al. (2018): one model where the allele frequency did not change over the transect and two other models where frequencies varied along the shore, one linearly and the other clinally. Five different cline shapes were compared: a simple symmetric cline, an asymmetric cline, a cline with a left tail, a cline with a right tail, or a cline with both tails, following the equations from Derryberry et al. (2014). In all fits, end frequency estimation was included; that is, variants were not assumed to be fixed different between ecotypes. A model with more parameters was considered to improve the fit if the differences in AIC were above four with the second best model. The goodness of cline fit was then assessed using a generalized linear regression model with binomial error to evaluate how well the frequency estimated from the cline explained the genotype observed for each snail given its position on the transect. For the inversion and the AK allele, we then calculated the deficit in heterozygosity (*F*_IS_) over seven bins of equal distances along each transect, using a custom R script developed in Reeves et al. (In prep.) that uses the results of the best‐fitting cline to infer the expected genotype frequency in each bin. The variation in snail size along the shore transects was also fitted to a simple symmetrical cline model, following Westram et al. (2018), and then compared to allelic clines. To facilitate the inference, and notably the estimates of confidence intervals, the width of the clines was log‐transformed, and the allele frequencies of the allelic clines were logit‐transformed.

##### 2.2.4.3 A temporal comparison using a previous study

The southern shore of the island was also studied by Tatarenkov and Johannesson (1999), and this offered a possibility to study the temporal dynamics of the genetic structure. The frequencies of the *Ak* alleles in 1998 were estimated at each of seven sampling sites along the transect. The position of each 1998 site on our one‐dimensional path was approximated by finding which of our 2018 snails was sampled closest to the same position. The transect position of this snail was then used to infer the pattern of the *Ak* variation observed in 1998 using the same cline functions as described above.

##### 2.2.4.4 Suspension bridge fitting

Differentiation between arrangements for an inversion is expected to take the form of a “suspension bridge” (Guerrero et al., 2012) with peaks of differentiation close to the breakpoints, where recombination is most strongly suppressed, and less differentiation in the center due to gene flux. We tested this expectation for the putative inversion on LG3 and used the resulting fit to ask whether the differentiation in the region of the arginine kinase locus was higher than expected given its position within the inversion. We approximated the suspension bridge pattern with a parabola inside the inversion and exponential declines outside, such that the expected differentiation, measured as *F*_ST_, took the following form:

$$F_{\mathrm{ST}.j}=m+a_{0}.e^{l*\left(p_{j}-b\right)};p_{j}<b$$

$$F_{\mathrm{ST}.j}=m+a_{0}+a_{1}\left(p_{j}-b\right)+a_{2}{\left(p_{j}-b\right)}^{2};p_{j}>b\wedge p_{j}<\left(b+k\right)$$

$$F_{\mathrm{ST}.j}=m+h.e^{r*\left(\left(b+k\right)-p_{j}\right)};p_{j}>\left(b+k\right)$$

where *m* is the background mean *F*_ST_ outside the inversion, *p*_*j*_ is the map position on LG3 of SNP (or contig) *j*, with the inversion starting at position *b* and having length *k*. To the left of the inversion, differentiation declines at rate *l* and to the right at rate *r*. At *b*, differentiation is elevated by *a*_0_. It then follows a parabola with parameters *a*_*i*_ (i = 0,1,2) to the end of the inversion at *b + k*, where the elevation is *h = a*_*0*_ *+ a*_*1*_ *.k + a*_*2*_ *.k* ^2^. Note that the positions *b* and *b + k* are maxima for the differentiation and may not correspond precisely to inversion breakpoints.

The *F*_ST_ values between homokaryotypes, averaged across SNPs within each contig, were fitted to this expectation using RStan. *F*_ST_ estimates were logit‐transformed to give an approximately normal distribution, and an additional parameter, *s*, was included for the standard deviation of residuals, which was assumed to be constant across the linkage group. A suspension bridge was considered to be present if the 95% posterior intervals for the *a*_*i*_ parameters did not include zero. We calculated standardized residuals for the observed *F*_ST_ values in order to define a putatively neutral envelope of differentiation from a typical suspension bridge format (standardized residuals <3, corresponding approximately to a cutoff for outlier detection of *p* = 0.01) and specifically asked whether contig 265, containing the *Ak* locus, was an outlier as expected if it is a direct target of selection.

## 3 RESULTS

### 3.1 Characterization of the *Ak* gene

Amplification and sequencing of the CDS of the *Ak* gene in six *L. saxatilis* snails revealed two alleles, designated *Ak* ^A^ and *Ak* ^B^. The complete CDS comprised 1053 bp and translated into a protein sequence of 351 amino acid residues that contained AK conserved domains and showed 83% amino acid identity to AK in the gastropod *Conus novaehollandiae* (ADK73590) and >70% identity to other mollusk species. Similar to other organisms, the *Ak* gene in *L. saxatilis* had six exons and five introns.

### 3.2 Variation in the coding sequences and phylogenic relationship among alleles

**FIGURE 1:**
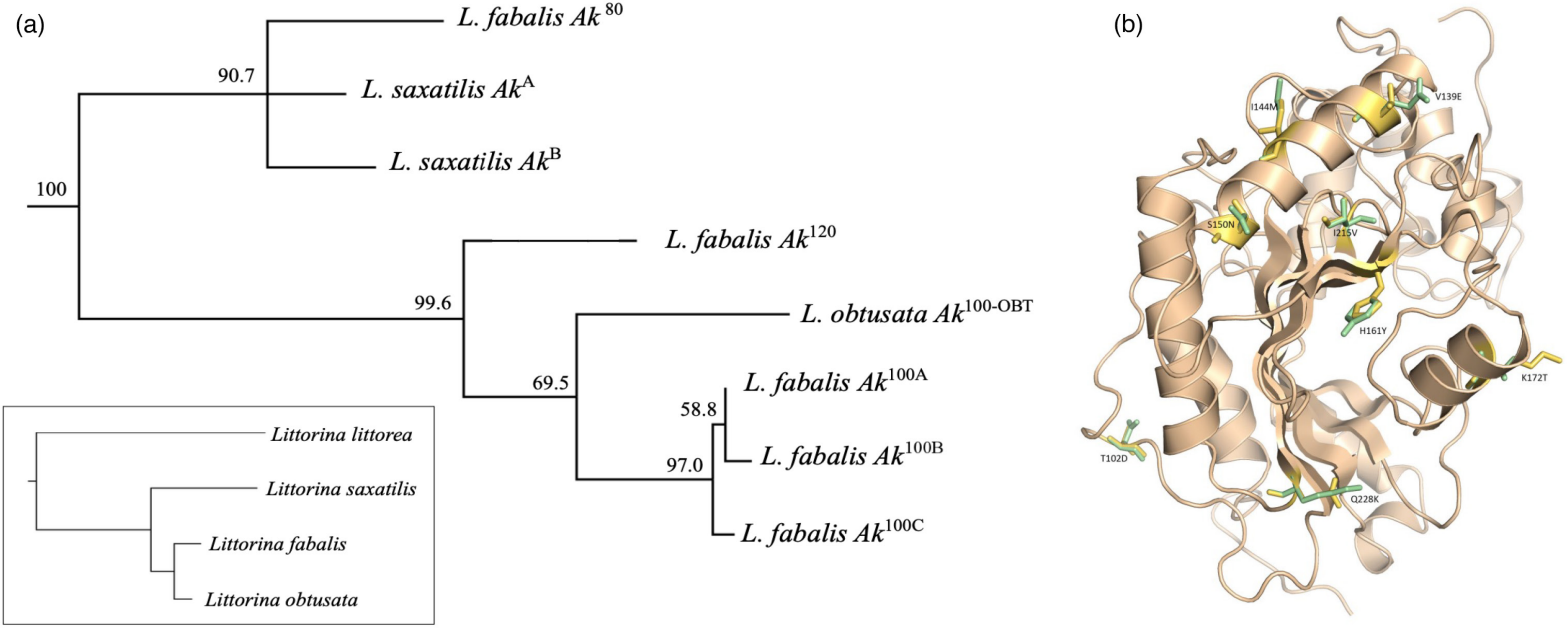
Haplotype variation in arginine kinase in individuals of *Littorina*. (a) A rooted neighbor‐joining gene tree showing the most likely relationships between *Ak* CDSs (1053 bp), using *C. novaehollandiae* (ADK73590) as the outgroup (not shown) and in comparison with the species tree from Reid et al. (2012) based on COI data (insert). (b) The illustration shows a 3D model of arginine kinase with the substitutions between *AK* ^100C^ and *AK* ^120^ illustrated as follows: XYYYZ, where X is the amino acid in *AK* ^100C^, YYY is the position of the substitution, and Z is the amino acid in *AK* ^120^ (see also Figure S1). The green and yellow colors illustrate the rough changes in 3D structure caused by the replacement where yellow corresponds to *AK* ^100C^ and green to *AK* ^120^

For *L. fabalis*, we obtained the full CDSs of the *Ak* ^120^, *Ak* ^100^, and the *Ak* ^80^ alleles. In addition, we sequenced partial transcripts (957 of 1053 bp and 319 of 351 amino acids) for 16 additional *L. fabalis* individuals, homozygous either for *Ak* ^100^ or for *Ak* ^120^ alleles. Based on partial transcripts, we found three different haplotypes of the *Ak* ^100^ allozyme allele (100‐A, 100‐B, and 100‐C) and a single haplotype of the *Ak* ^120^ allele. None of the individuals had more than two haplotypes of the *Ak* coding sequence. The *Ak* ^120^ haplotype and the most common of the *Ak* ^100^ haplotypes (*Ak* ^100‐C^) differed by 10 substitutions, eight of which were nonsynonymous, while *Ak* ^120^ and the two other *Ak* ^100^ haplotypes (A and B) differed by seven amino acids (Figure S1). These substitutions are predicted to cause differences in protein charge and the isoelectric points, reflecting well the expected changes in electrophoretic mobility previously observed on the allozyme gels (Table S1). The *Ak* ^80^ allele differed from both *Ak* ^100^ and *Ak* ^120^ by 11 nonsynonymous mutations, all of them shared with *L. saxatilis*, and *Ak* ^80^ and all three *Ak* ^100^ haplotypes differed in four additional positions also shared between *Ak* ^80^ and *Ak* ^120^ (Figure S1). Finally, the *Ak* ^100^ allele in *L. obtusata* differed from the *Ak* ^100^ in *L. fabalis* by 7–8 nonsynonymous mutations (Figure S1) but shared the same isoelectric point (Table S1). Altogether, the *Ak* gene tree shows a deep divergence of the alleles found in *L. fabalis* (Figure 1a). The 3D model of AK suggests that all amino acid replacements between *L. fabalis Ak* ^100^ and *Ak* ^120^ alleles are positioned on the surface of the globular enzyme (Figure 1b).

### 3.3 Genomic context and population structure around the *Ak* gene

The *Ak* transcript sequences mapped to contig 265 of the *L. saxatilis* reference genome (Westram et al., 2018), with the *Ak* locus spanning positions from about 20 to 67 kb within this contig. Exons 1, 3, and 4 were represented in two or three copies on contig 265, and exon 6 was also partially duplicated on the same contig (Figure S2a), suggesting either a duplication of the gene or errors in the current genome assembly (Figure S2b). In addition, we found increased coverage of the regions including *Ak* exons 5 and 6 in the individuals sequenced at higher coverage (15×), suggesting the presence of multiple copies of part of the gene in *L. fabalis* individuals (Figure S3).

**FIGURE 2:**
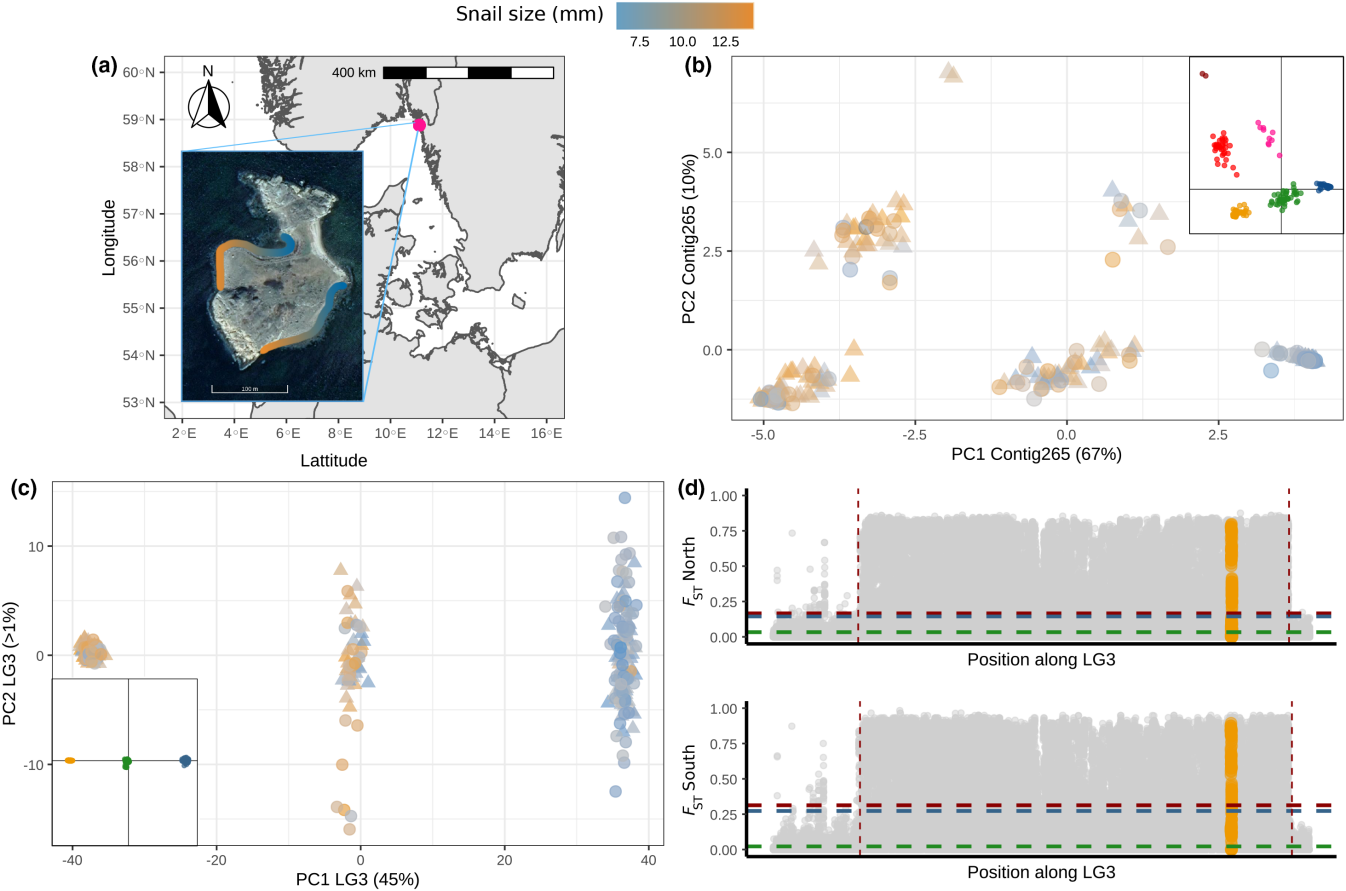
Sampling location and population structure of two 175‐m‐long *L. fabalis* transects. (a) The location of Lökholmen with a picture of the island. The northern and southern transects are highlighted with a color gradient that corresponds to the gradients of snail size sampled (as indicated above the plot). (b) A PCA performed on the 70 SNPs from contig 265 containing the *Ak* gene showing six clusters as expected from combination of three divergent haplotypes into different genotypes. (c) A PCA performed on 9905 SNPs from the overall linkage group pruned for closely linked SNPs (LG3 of the *L. saxatilis* reference map) with three distinct clusters suggesting the presence of an inversion. In (b) and (c), each point corresponds to an individual snail (triangles represent northern transect, and circles represent southern transect) filled by a colored gradient proportional to the snail's size. Insert in b and c shows the outcomes of a DAPC analysis based on clusters inferred from find.clusters in adegenet for *k* = 6 and *k* = 3, respectively. (d) *F*_ST_ variation over LG3 estimated between snails from the ends of the northern (top) and southern (bottom) transects. Each gray dot corresponds to the *F*_ST_ value estimated for one of the 58,246 linked SNPs and sorted according to their rank position on the *L. saxatilis* linkage map. Contigs are ordered by position on the linkage map, with random orientation and random order within map positions. SNPs within the contig 265 are highlighted in orange. The horizontal broken lines show the average *F*_ST_ values over the entire LG (blue), outside the inversion (green) and inside the inversion (red). The limits of the inversion were defined by eye and are shown on the graphs by the two vertical dashed lines

The assembly errors and potential duplications led to problems associated with the SNP calling, explaining why none of the SNPs called with our pipeline were inside the *Ak* exons. Yet, we were able to identify 70 SNPs distributed across contig 265 (Figure S4). A PCA on these 70 SNPs showed six distinct groups of individuals (Figure 2a,b) forming a “triangle” on the PCA. Three groups were located in the triangle's corners, and the three remaining groups were positioned at the midpoints of each side of the triangle. The subset of SNPs in the upper 5% eigenvalue quantile, which contributed most to the sample dispersion along PC1 and PC2, were distributed over the entire length of contig 265 (Figure S4), in regions without any issues in the current assembly and located between the *Ak* exons. The clustering analyses performed through DAPC captured the subdivision in six clusters over contig 265 (insert in Figure 2b). Observed heterozygosity was much higher (0.31 < *H*_obs_ <0.38) in the clusters located in the midpoints of the sides, than in the corner clusters (0.04 < *H*_obs_ <0.05). These observations are consistent with the presence of three divergent haplotypes for this contig, and their corresponding six genotypes, three homozygotes in the corners with heterozygotes placed in between (Figure 2b).

### 3.4 Population structure based on LG3

A PCA performed using the 9905 SNPs (pruned for close linkage) from the entire LG3 of *L. fabalis* showed similar population structure in the two studied transects. Most of the large individuals sampled along the wave‐exposed parts of the transects (orange in Figure 2a,c) were separated from most dwarf individuals sampled along the sheltered part (blue in Figure 2a,c) along the first axis, which explained 45% of the total variation. A third group composed of both ecotypes and putative hybrids of intermediate size was localized in the center. The second axis, showing less than 1% of the variation, reflected minor genetic variation among, mainly, dwarf individuals. Clustering analyses using DAPC captured the subdivision into three clusters (insert Figure 2c). The observed heterozygosity was lower among individuals located on the left side of the PCA (the majority of which were large individuals; mean *H*_obs_ = 0.09) than among individuals on the right side of the graph (mostly dwarf individuals; *H*_obs_ = 0.12). Individuals from the center of the PCA showed heterozygosity twice as high as the other groups (*H*_obs_ = 0.24) and exhibited many more SNPs with high values of *H*_obs_ (1946 vs. 31 or 24 SNPs with *H*_obs_ >0.80; Figure S5). Taken together, these patterns likely reflect the presence of a large chromosomal rearrangement, such as an inversion, with the three clusters representing homokaryotypes for the two arrangements (right and left clusters) and heterokaryotypes (in the middle; Figure 2c).

Pairwise *F*_ST_ values calculated between snails from the ends of the two transects representing the two different ecotypes clearly show the presence of a large genomic island of divergence along LG3 (Figure 2d), most likely corresponding to a chromosomal inversion. High values of *F*_ST_ (above 0.8) and LD (Figure S6) characterized the entire inversion. Average *F*_ST_ and LD estimated along the entire LG were high (*F*_ST_ = 0.27, *r* ^2^ = 0.127), and an order of magnitude was higher within the putative inversion (*F*_ST_ = 0.32, *r* ^2^ = 0.168) than outside (*F*_ST_ = 0.02, *r* ^2^ = 0.005). We found 484 SNPs (1% of the dataset unpruned for linkage) fixed for different alleles when comparing the two homozygote groups of the overall PCA (Figure 2c). The 71‐kb‐long contig 265 carrying the *Ak* gene was localized within the inversion (orange points in Figure 2d), approximately 12 cM away from the nearer breakpoint based on the *L. saxatilis* linkage map (Westram et al., 2018), and showed an average *F*_ST_ value of 0.31 between ecotypes.

### 3.5 Suspension bridge and outlier test

**FIGURE 3:**
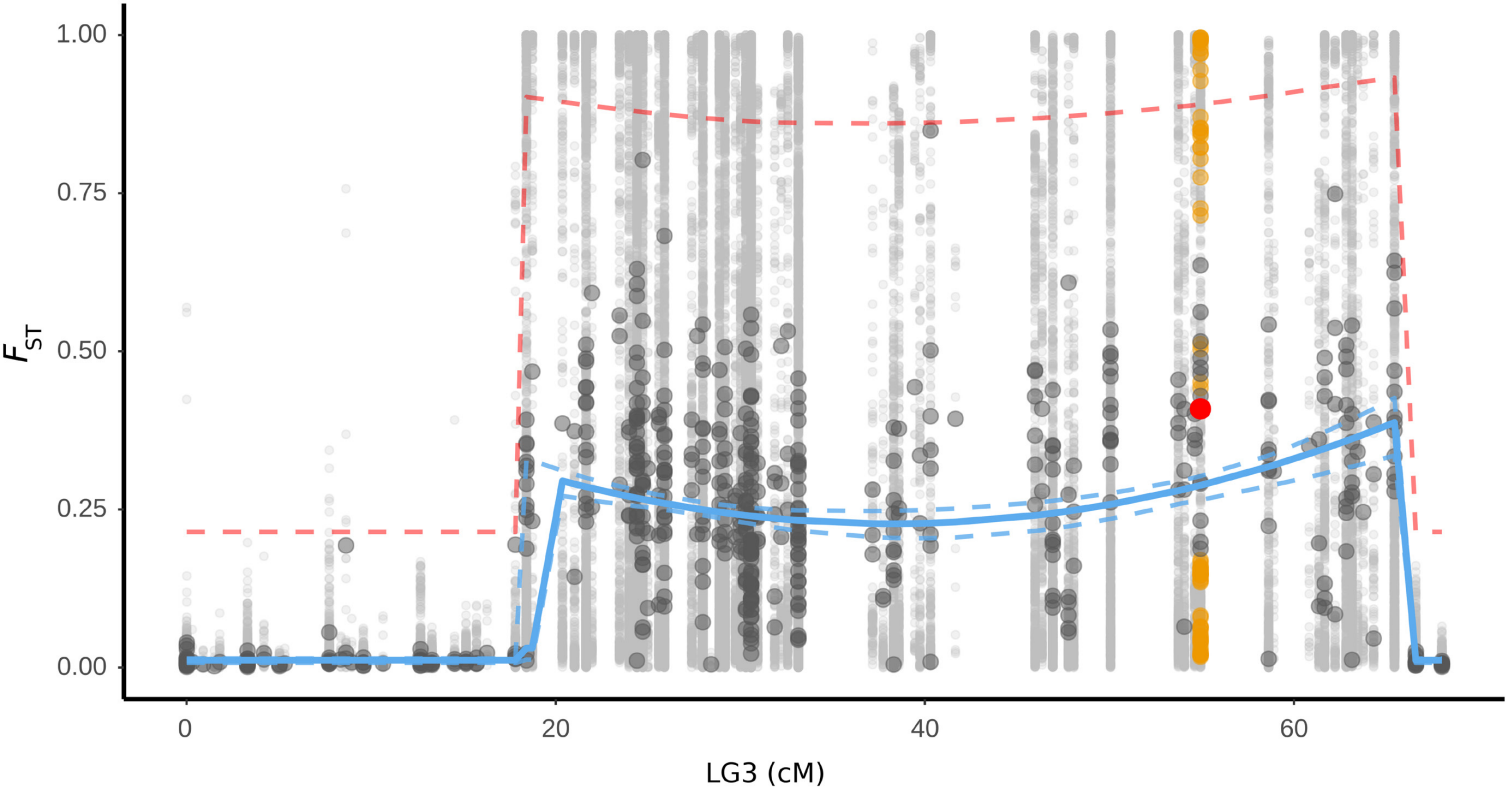
Suspension bridge analysis performed on all homokaryotype individuals from both transects. The large dark gray dots represent average *F*_ST_ values by contigs, and the small light gray dots represent *F*_ST_ of individual SNPs sorted based on their average positions on the *L. saxatilis* linkage map (Westram et al., 2018). Average and SNP *F*_ST_ values from the contig 265 carrying the *Ak* locus are highlighted in red and orange, respectively. The solid blue line corresponds to the fit of a suspension bridge model on the variation of mean *F*_ST_, performed in RStan using the equations detailed the Methods section, and the dashed blue line corresponds to the 95% confidence interval of the average *F*_ST_. The dashed red line represents the outlier detection threshold for contigs (three standard deviations above the fitted mean *F*_ST_)

As expected given the recombination patterns within inversions (Guerrero et al., 2012), the landscape of differentiation within the putative inversion followed a “suspension bridge” pattern (Figure 3) with *F*_ST_ significantly lower in the center of the inversion than close to the breakpoints, and the confidence intervals of the parabola parameters (a_0_, a_1_, and a_2_) were all different from zero (Table S2). The average *F*_ST_ value at the *Ak* contig (red dot, Figure 3) was above the average *F*_ST_ value expected from the suspension bridge (blue line) but below the outlier detection threshold (dashed red line, >3 standard deviations from the mean on the logit scale). Indeed, we could not detect any contig on LG3 that was an outlier compared to the suspension bridge expectation.

### 3.6 Relationship between inversion genotypes and the *Ak* alleles

As the structure patterns in the whole LG3 and on contig 265 likely reflect the presence of a large inversion carrying the *Ak* gene and three divergent haplotypes at this gene, these clusters were used to assign each snail to an inversion karyotype and *Ak* genotype, respectively (Table 1). We named the arrangements based on their frequency in each ecotype, that is, the arrangement L being found in high frequency in the exposed part of the shores in mostly large snails, and the arrangement D being found in high frequency in the sheltered part of the shores in mostly dwarf snails. The homokaryotype individuals carrying two L‐ or two D‐arrangements were therefore assigned as L/L or D/D, respectively, and heterokaryotype individuals were assigned as L/D. All the D/D individuals (right cluster in Figure 2c, in blue) were assigned to the same cluster in the DAPC from contig 265 (Table 1), and we infer that this corresponds to homozygotes for the *Ak* ^120^ allele based on the observed frequencies in the transects from the previous allozyme studies (Tatarenkov & Johannesson, 1999). The L/L individuals were assigned to three *Ak* genotypes, two of which most likely correspond to homozygotes for *Ak* ^*80*^ (here found in low frequency in the large ecotype as in earlier allozyme studies) and *Ak* ^*100*^ (found in high frequency in the large ecotype), and the last L/L being the heterozygotes *Ak* ^*80*^/*Ak* ^*100*^. Finally, the heterokaryotype individuals (L/D) were composed of the two *Ak* heterozygous genotypes *Ak* ^*100*^/*Ak* ^*120*^ and *Ak* ^*80*^/*Ak* ^*120*^. Overall, the *Ak* alleles carried by different arrangements lead to a strong correlation between the positions of the snails in the PCA performed on SNPs from contig 265 and from the whole LG3 (Figure S8). Altogether, this suggests that the L‐arrangement exclusively carries the *Ak* ^*120*^ allele, and the D‐arrangement carries the alleles *Ak* ^*80*^ and *Ak* ^*100*^. Surprisingly, the average heterozygosity of the heterozygote *Ak* ^80^/*Ak* ^100^ from the same arrangement was even higher than the average heterozygosity of the heterozygote Ak^100^/*Ak* ^120^ found in heterokaryotype individuals (Table 1).

**TABLE 1:** *Ak* genotypes inferred from the genetic clusters in Figure 2b

Contig 265 clusters	*N*	*H*_obs_ *Ak*	L/L	L/D	D/D
80/80	2	0.05	2	0	0
80/100	34	0.38	34	0	0
80/120	10	0.33	0	10	0
100/100	82	0.05	82	0	0
100/120	44	0.31	0	44	0
120/120	123	0.04	0	0	123

*Note*: In order of appearance, the number (*N*) of individuals assigned to each putative genotype, observed heterozygosity calculated using SNPs from contig 265 (*H*_obs_ *Ak*), and number of individuals with a given *Ak* genotype found in each inversion cluster (from PCA in Figure 2c) are shown.

### 3.7 Clines along the seashore

**FIGURE 4:**
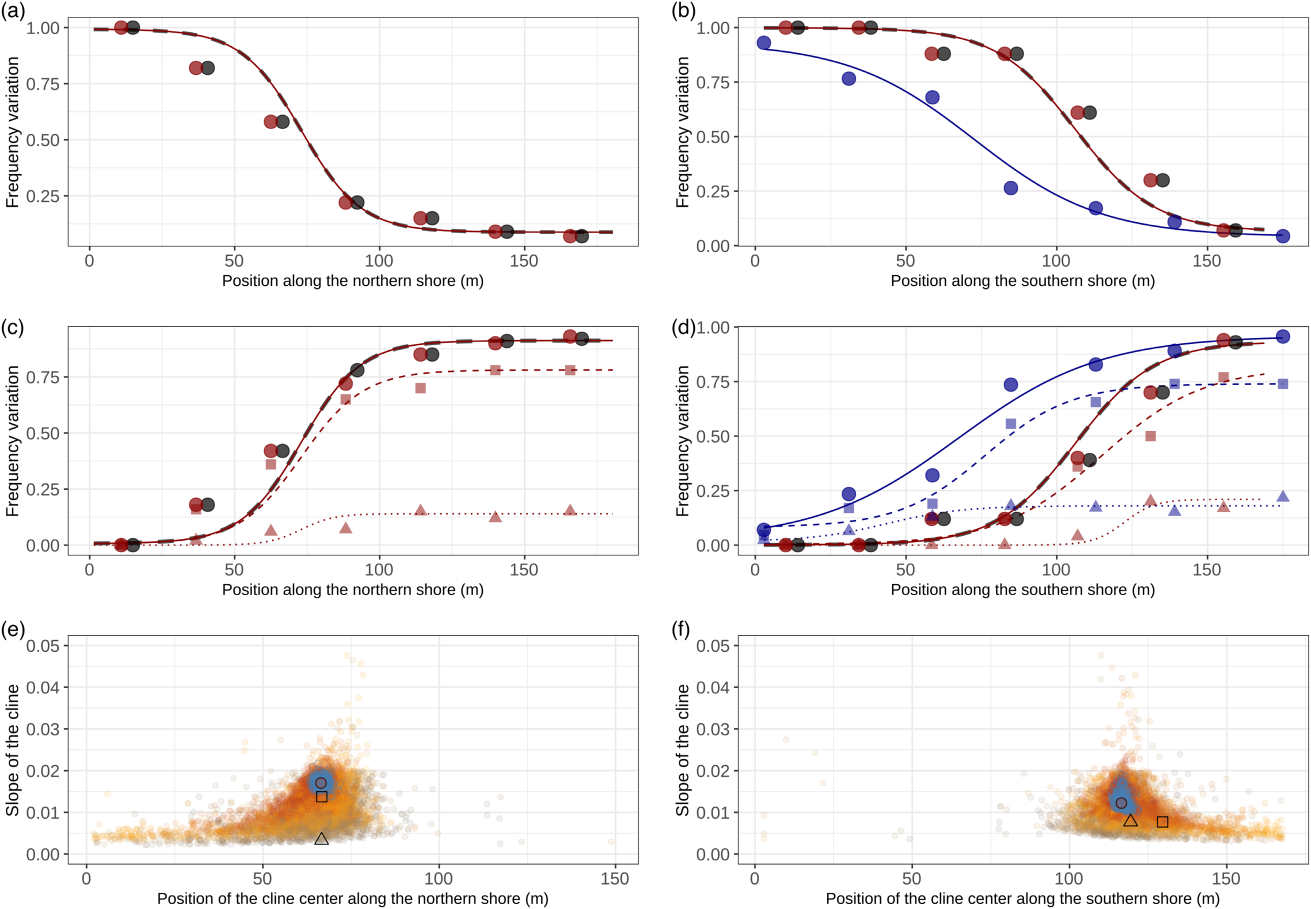
Frequency clines across the northern (a, c, e) and the southern transects (b, d, f). In (a–d), the dashed black lines show the arrangement cline at the putative inversion inferred from the three clusters of the PCA in Figure 2c, and the dark red lines are the cline fits for the three major alleles of the *Ak* gene inferred from the six clusters in Figure 2b. Large dots are the arrangement and allele frequencies estimated for seven equal distance bins along each transect (black showing frequencies of one inversion arrangement and red showing the *Ak* allele frequencies). Dark blue dots and lines correspond to the allele frequency clines inferred from the allozyme data for the southern transect of Tatarenkov and Johannesson (1999). In (e–f), the graphs represent the slope of cline (differences of allele frequency divided by the width of the cline) against the center of the cline estimated for each SNP with a significant clinal variation. The color gradient (from gray to orange) shows the frequency difference between the ends of the transect (from 0 to 1). Differentially fixed SNPs between arrangements are highlighted in blue. In a–f, circles are *Ak* ^*120*^; triangles, *Ak* ^100^; and squares, *Ak* ^*80*^

On both transects, frequency variation in the inversion arrangements and the *Ak* alleles estimated from the clustering analyses were also best described using the “simple cline” model, with only one minor exception for the Ak^80^ allele in the southern transect (Table S3). The inversion arrangement and *Ak* allele cline centers (from the clustering analyses) were close to the centers of the phenotypic clines in both transects, as evident from overlapping 95% confidence intervals (Table S4). The *Ak* ^*120*^ cline perfectly overlapped with the D‐arrangement cline (Figure 4), while the sum of the frequencies of *Ak* ^*100*^ and *Ak* ^80^ showed a 1:1 correspondence with the cline observed for the L‐arrangement (Figure 4c,d), as expected from the clustering analyses (Table 1). In addition, we found 12,386 SNPs (18 inside contig 265) and 10,909 SNPs (31 inside contig 265) showing evidence for clinal variation along the northern and southern transect, respectively (i.e., SNPs with allele frequency cline fit with a difference of AIC > 4 with linear or stable allele frequency fit). Furthermore, 99% of the clinal SNPs were located inside the inversion (limits taken from the suspension bridge, Table S2). The frequency at the *Ak* ^*120*^ and the *Ak* ^*80+100*^ showed similar clines to the 484 SNPs that are also fixed different between inversion arrangements, closely following the arrangement clines as expected (showed by the slope of the cline in Figure 4e,f, and by the results of the cline fit in Figure S9). These SNPs were found over the entire inversion (Figure 3, individual loci with *F*_ST_ = 1). All of these clines had high goodness‐of‐fit scores (Figure S10). This goodness of fit has been shown to be a good proxy for detecting markers directly or indirectly affected by selection (Westram et al., 2018). Overall, this suggests that signs of selection are widespread across the entire inversion.

In both transects, the *Ak* ^*120*^ allele and the D‐arrangement were close to fixation in the most sheltered part of the shore (dwarf ecotype) but remained at low frequency in the exposed part (<0.1), leading to a slight excess of heterozygote (−0.18 < *F*_IS_ < −0.08; Table S5). This contrasted with the middle of both transects where a deficit in heterozygotes was found (*F*_IS_ ranging from 0.07 to 0.70; Table S5) where dwarf and large ecotypes overlap in distribution (Figures S11 and S12). However, most of the *F*_IS_ values (except one that was significant without Bonferroni correction) had nonsignificant *p*‐values despite high *F*_IS_ values and more samples are needed to properly test their significance (Table S5).

## 4 DISCUSSION

The strong size dimorphism with a large and a dwarf ecotype of *L. fabalis* (Reimchen, 1981) in northern and western European populations, and its strong association with variation in the arginine kinase gene, has been the focus of several earlier studies (e.g., Kemppainen et al., 2005, 2011; Tatarenkov & Johannesson, 1994, 1998, 1999). However, the question of whether this differentiation was caused by divergent selection targeting the *Ak* locus or nearby loci, or other processes, has remained unanswered. Here, we use a new toolbox to characterize the molecular and genomic background of the polymorphism in arginine kinase and its strong association with ecotype divergence in *L. fabalis*. By first dissecting the nucleotide polymorphism in the CDS of the *Ak* locus, we found that the three main alleles identified in earlier allozyme studies differ by a large number of nonsynonymous mutations. Notably, the net differences in protein charge resulting from these substitutions fully explain the different migration of the allozyme alleles in starch gel electrophoresis and, in addition, show that the *Ak* locus itself remains a strong candidate for being under selection. Moreover, using WGS, we also found that the *Ak* locus is embedded in a putative large chromosomal inversion and may be affected by introgression and by other types of structural variations, likely including at least partial gene duplication.

### 4.1 Origins of the variation observed at the *Ak* locus

#### 4.1.1 A large chromosomal inversion

The karyotype of different species within the genus *Littorina* is highly conserved (García‐Souto et al., 2018). Hence, we could map the *L. fabalis* sequences onto the *L. saxatilis* reference genome and genetic map. Doing this, we found a large region of LD around the *Ak* gene (Figure S6) located on LG3 (affecting 47 cM of the 69 cM LG3 from the *L. saxatilis* map), with genomics signatures expected from a chromosomal inversion (Cayuela et al., 2021; Le Moan et al., 2021; Mérot et al., 2020). This putative inversion covers around ~4.7% of the reference genome and contains 1053 of the ~13,000 genes positioned on the linkage map (Westram et al., 2018). However, as the current assembly is estimated to contain around ~25,000 genes of which half remains to be map, this is probably an underestimation of the gene content of the inversion. The presence of a putative inversion containing the *Ak* locus fits the observation of high LD (0.8) between the allozyme polymorphism and the anonymous genetic marker described by Johannesson and Mikhailova (2004) and the strong association between shell size and *Ak* genotype (Tatarenkov & Johannesson, 1998). This putative inversion carries most of the genetic differences between *L. fabalis* ecotypes present in LG3, while the collinear part of this LG remains mostly undifferentiated, as observed in other species (Cheng et al., 2012; Kulathinal et al., 2009). The reduction in *F*_ST_ near the center of the inversion shows that a significant amount of gene flux is likely occurring in this region, as expected from a large inversion, due to double crossover and gene conversion (Guerrero et al., 2012; Kirkpatrick, 2010). Although chromosomal inversions play a significant role in ecotype formation in *L. saxatilis* (Faria et al., 2019; Koch et al., 2021; Morales et al., 2019; Westram et al., 2021), no inversion has so far been detected in LG3 in *L. saxatilis* (Faria et al., 2019), and the two alleles that we found at the *Ak* locus in *L. saxatilis* based on the six individuals here sequenced were not strongly divergent. This suggests that the LG3 inversion in *L. fabalis* is not ancestral to the clade. In addition, the most frequent arrangement in large individuals (L) shows less diversity than the one frequent in the dwarf individuals, suggesting that the L‐arrangement is the derived form of the inversion. However, this low diversity could also reflect recent fluctuations in effective population size in the large ecotype (discussed below). Therefore, further studies exploring the phylogeny of the inversion's breakpoints among Littorinidae are needed to fully understand the history of this inversion.

#### 4.1.2 A partial duplication

We also found increased coverage on parts of contig 265 within the inversion carrying the *Ak* locus, compatible with a partial duplication of *Ak* (Figure S3), as already suggested from intron sequencing of *Ak* (Kemppainen et al., 2011). We did not detect duplicates in our transcript sequencing, and the fragmentation and possible errors in the current genome assembly make it difficult to evaluate this hypothesis. The nature of any duplication, whether it is expressed, and its potential consequences for the gene expression of *Ak* and functional effects on ecotype trait differentiation will require further study (cf. Berdan et al., 2021).

#### 4.1.3 Potential introgression

While the WGS only focused on *Littorina fabalis*, our phylogenetic analyses based on the exon sequencing included two additional species from the *Littorina* genus: *L. saxatilis* and *L. obtusata*. This phylogeny shows that the *Ak* ^*80*^ allele found in *L. fabalis* individuals is genetically close to the alleles found in *L. saxatilis*, the most divergent species included in the analyses. This surprising discordance between gene and species trees at the *Ak* ^*80*^ allele, and its presence in populations of *L. fabalis* sampled in eight Swedish islands (Tatarenkov & Johannesson, 1994) while absent in samples from France (*n* = 63) and Wales (*n* = 24; Tatarenkov and Johannesson, unpubl. allozyme data available in Table S6), suggests that this allele has recently introgressed from *L. saxatilis* into *L. fabalis*. This introgression between divergent species would also explain why the heterozygote *Ak* ^*80*^/*Ak* ^*100*^ showed the highest observed heterozygosity (0.38) among the contig 265 clusters (Table 1). *Ak* ^*80*^ is only found in the L‐arrangement, and in the south shore at Lökholmen, its frequency (~19%) appears to be relatively stable over time (Table S4), although allozyme data suggest its frequency is variable among wave‐exposed sites (0.03–0.40; Tatarenkov & Johannesson, 1994). A higher taxonomic sampling and a more contiguous genome assembly are needed for further investigation of the history of this allele.

### 4.2 Temporal variation in cline position

Variation in the *Ak* gene along the southern transect of Lökholmen was mapped using allozymes 23 years ago (Tatarenkov & Johannesson, 1999). When we compared the shape and position of the *Ak* cline between the two time points, we found that, while our allele frequency estimates toward the ends of the transect were similar between studies, the cline center had shifted ~30 m toward the more exposed part of the shore in 2018 compared to 20 years earlier (Figure 4). The shifted cline center is quite intriguing, but one speculative interpretation is that the shift is a consequence of large individuals being nearly depleted and partly replaced by dwarf individuals during the period 2000–2010, due to intense scientific sampling (KJ pers. obs.). Sampling along this shore was stopped in 2010, and in 2018, the large ecotype had re‐established at densities similar to those in pre‐2000 samples. Other factors could also explain this apparent spatial instability of the genetic cline, such as interannual variation in the exposure gradient, or temporal variation in ecotype reproduction patterns of this annual species (Williams, 1992).

### 4.3 Heterozygote deficiency at the cline centers

A strong deficit of heterozygotes was observed in the allozyme study for subsamples containing both large and dwarf individuals (Tatarenkov & Johannesson, 1999). The same deficit is suggested, albeit not significant, in the cline center for the *Ak* allele and the arrangement frequencies inferred from the PCA (Table S5). The smaller sample sizes in our study than in Tatarenkov and Johannesson (1999) may be a reason for a weaker trend in our data. Size assortative mating (Saltin et al., 2013) and/or low fitness of hybrids due to maladaptation and/or genetic incompatibility can explain such a deficiency.

### 4.4 Evidence of selection on *Ak*

The inversion, like the *Ak* alleles, reaches near differential fixation across the two studied transects. The sharp allelic frequency cline inferred over such a small geographical scale in both transects, despite the numerous heterozygotes observed in the center of the clines, shows that selection must be involved (Haldane, 1948). Field estimates of snail dispersal distances (Tatarenkov & Johannesson, 1998) suggest 1–2 m over a month for the majority of snails but displacement of 10 m for single snails after 2 months. Using an estimate of lifetime (12 months) mean dispersal distance (σ) of 10 m, and a cline width of 65 m (average over the two transects; Table S3), selection on the inversion, s*, is in the range of 0.07 (using width = 1.732 σ/√s*, assuming an abrupt habitat change and no dominance; Barton & Gale, 1993). However, this estimate is sensitive to the dispersal value, σ, and with a σ of 20 m, estimated selection instead becomes 0.11 on the inversion. Even if relatively strong selection is shaping the inversion cline, the *Ak* locus can be either the single or more likely one of several targets of selection, or through linkage to all other loci of the inversion, instead being hitchhiked with selected loci within the inversion (Cheng et al., 2012). Indeed, we found 484 SNPs differentially fixed between arrangements (and therefore close to fixation between ecotypes) distributed throughout the entire inversion. As expected, these SNPs showed strong and closely similar clinal frequency variation along both transects (Figure S9). These fixed differences suggest that the inversion is rather old. Given the evidence for gene flux between rearrangements from the suspension bridge pattern, at least a proportion of them are likely to be maintained in complete association with the arrangements by selection, and therefore, they are candidates for contributing to the adaptive significance of the inversion polymorphism. The *Ak* ^*120*^ allozyme allele falls into this category since it is fixed on the D‐arrangement and absent on the L‐arrangement. However, the SNPs in exons responsible for the allozyme variation were not included in the set of SNPs analyzed in contig 265, perhaps explaining why this contig, which contains the *Ak* gene, was not an outlier in the suspension bridge analysis. As the *Ak* locus is likely ~12 cM away from the nearest breakpoint, more data from additional hybrid zones and more SNPs positioned on an improved genome assembly will be needed to determine whether the differentiation in the *Ak* gene is more resistant to gene flow than nearby regions in the inversion, which would support selection on this gene.

From the gene characterization, we found that the two dominant AK allozymes in *L. fabalis* differed by 7–8 amino acid substitutions, while in most cases, protein polymorphisms under selection include one or a few amino acid substitutions (Nielsen, 2005). The 3D model suggested that these substitutions were positioned on the surface of the AK enzyme and not in the active site. Mutations at the surface are likely to improve enzyme properties without impeding the function, for example, by altering the affinity to substrate molecules (Storz & Wheat, 2010; Wheat et al., 2006). That is, the many mutations may be involved in modulating the rate of enzyme activity between the AK100 and AK120 allozymes, or they may be involved in epistatic effects through interactions with other proteins. Altogether, this suggests that *Ak* remains a strong candidate gene for being a target of selection, possibly as part of a co‐adapted set of genes inside the inversion.

## 5 CONCLUSION

By exploring the genomic variation at and around the *Ak* gene in *Littorina fabalis*, we discovered that divergent *Ak* alleles are found on two different arrangements of a large inversion. These arrangements reach near differential fixation at the ends of short transects crossing environmental discontinuities that correspond to hybrid zones between a large and a dwarf ecotype of *Littorina fabalis*. This observation adds to the growing body of evidence describing chromosomal inversion polymorphisms associated with genetic differences across environmental gradients (Wellenreuther & Bernatchez, 2018). The presence of an inversion shows that *Ak* is part of a genomic barrier extensively larger than that of a single selected locus and its linked genomic region. Many SNPs within this inversion showed similar trends, being differentially fixed between arrangements, and showing clear allelic frequency clines along the transects. This suggests that signs of selection are found over the entire inversion despite the evidence of gene flux between arrangements, and this also weakened our ability to detect which loci inside the inversion are the actual targets of the divergent selection. Here, the *Ak* locus is among the clinal marker observed to be fixed different between inversion arrangements and the high number of nonsynonymous replacements illustrates important differences between the *Ak* alleles. Taken together, the *Ak* gene still seems likely to contribute to the differential selection that maintains the strong inversion clines in both shores, but it will require further experiments to confirm this hypothesis. This study illustrates how a new sequencing toolbox can be used to characterize the genomic architecture associated with classical candidate genes.

## CONFLICT OF INTEREST

The authors declare no conflict of interest.

## Supporting information


Appendix S1

## Data Availability

The different haplotype sequences found at the AK gene are available in GenBank under the accession numbers OM938024 to OM938031. The raw fasta files are available on NCBI SRA under the project name “Littorina fabalis transect WGS” with accession number PRJNA836378 after 6 months of embargo. The three vcf files used for the analyses, which contain respectively the 70 SNPs from the contig 265, the 58,246 SNPs unpruned for linkage, and the 9905 SNPs pruned for linkage from the LG3, as well as the R script used to analyze these datasets, are available as zenodo archive (https://doi.org/10.5281/zenodo.6482922).
