# Supplementary material for: An allozyme polymorphism is associated with a large chromosomal inversion in the marine snail Littorina fabalis
Source: Evol Appl. 2022 Jun 26;16(2):279–92. doi: 10.1111/eva.13427 (PMC9923470; doi:10.1111/eva.13427)
Supplement: Supplementary file 1 — Appendix S1 [file EVA-16-279-s001.pdf]

Supplementary material

Table S1. Estimated isoelectric points of the AK allozymes of *Littorina* at a pH=8.6. The differences in electric charge of the allozymes of AK correspond well to their relative mobility during electrophoresis, where the names of the alleles indicate their relative mobility, that is the allele Ak^{120} produced an allozyme that moved 20% further than the reference allozyme (coded by the Ak^{100} allele) and Ak^{80} is the allele for the allozyme that moved 20% shorter distance.

**Species**	**Allele**	**Isoelectric point**
L. fabalis	Ak^{120}	6.68
L. fabalis	Ak^{100}	7.10
**L. fabalis**	Ak^{80}	7.61
L. obtusata	$Ak^{100\text{-OBT}}$	7.10
L. saxatilis	Ak^{A}	7.93
L. saxatilis	Ak^{B}	7.31

Table S2: Results of the suspension bridge fitting for the samples used to calculate the F_{ST} between all homokaryotypes, and between snails from transect ends (North and South). Mean and confidence intervals [upper CI – lower CI] are indicated for F_{ST} values outside the inversion (m), the linkage map position of the first breakpoint (b), the inversion length (k), the standard deviation of mean F_{ST} value (s, on logit scale), the elevation of F_{ST} at b (the start of the inversion; a_0), the parameters of the parabola (a_1 and a_2).

Data set	m	b	k	s	a_0	a_1	a_2
Homokaryotypes	0.0108 [0.0081 - 0.0137]	18.86 [17.85 - 20.95]	47.02 [44.78 - 48.42]	1.15 [1.07 - 1.22]	0.236 [0.213 - 0.286]	-0.00551 [-0.01186 - -0.00286]	0.00016 [0.00009 - 0.00030]
North	0.0115 [0.0098 - 0.0132]	17.98 [17.82 - 18.36]	48.32 [47.46 - 48.58]	0.94 [0.92 - 0.97]	0.177 [0.137 - 0.209]	-0.00552 [-0.00831 - -0.00047]	0.00013 [0.00002 - 0.00019]
South	0.0167 [0.0118 - 0.0194]	18.68 [17.84 - 20.35]	46.97 [45.19 - 48.32]	1.10 [1.04 - 1.17]	0.275 [0.234 - 0.312]	-0.00326 [-0.00632 - -0.00107]	0.00010 [0.00004 - 0.00018]

Table S3: Comparison of the fit obtained from seven models (Simple, Asymmetric etc.) of allele frequency variation along the two transects (South and North). The goodness of fit is compared with the AIC value, and the differences between the AIC of a given cline model to the best fitted cline (Δ_{AIC}) are indicated. The clines were fitted using the arrangement karyotype (Arrangement) and *Ak* genotype (Ak^{120}, Ak^{100}, Ak^{80}) obtained from the clustering analyses (see Method) and include also a fit where the two alleles located on the same arrangement were pooled (Ak^{80+100}).

Transect	Fitted data	Model choice	Simple	Asymmetric	Left tailed	Right tailed	Two-tailed	Linear	Flat
South	Arrangement	AIC	**187.57**	188.96	190.84	191.58	195.10	217.04	312.99
		Δ_{AIC}	**0.00**	1.38	3.27	4.00	7.52	29.46	125.42
	Ak^{120}	AIC	**187.57**	188.95	190.99	191.58	195.44	217.04	312.99
		Δ_{AIC}	**0.00**	1.38	3.42	4.00	7.86	29.46	125.42
	Ak^{100}	AIC	**200.29**	202.36	204.21	204.63	209.18	213.31	277.95
		Δ_{AIC}	**0.00**	2.07	3.92	4.34	8.89	13.03	77.67
	Ak^{80}	AIC	**83.97**	84.62	87.99	87.03	91.03	94.59	107.32
		Δ_{AIC}	**0.00**	0.65	4.01	3.06	7.06	10.62	23.35
	Ak^{80+100}	AIC	**187.57**	188.96	190.84	191.58	195.10	217.04	312.99
		Δ_{AIC}	**0.00**	1.38	3.27	4.00	7.52	29.46	125.42
North	Arrangement	AIC	**244.64**	246.97	248.67	248.54	252.57	285.23	409.12
		Δ_{AIC}	**0.00**	2.33	4.03	3.90	7.93	40.59	164.48
	Ak^{120}	AIC	**244.64**	246.96	248.65	248.37	251.21	285.23	409.12
		Δ_{AIC}	**0.00**	2.32	4.01	3.73	6.57	40.58	164.48
	Ak^{100}	AIC	**283.64**	285.61	287.68	287.45	291.71	303.19	395.60
		Δ_{AIC}	**0.00**	1.97	4.03	3.81	8.07	19.55	111.96
	Ak^{80}	AIC	**158.05**	160.08	162.06	161.57	165.62	**157.33**	167.77
		Δ_{AIC}	**0.72**	2.74	4.72	4.24	8.29	**0.00**	10.44
	Ak^{80+100}	AIC	**244.64**	246.97	248.67	248.54	252.57	285.23	409.12
		Δ_{AIC}	**0.00**	2.33	4.03	3.90	7.93	40.59	164.48

Table S4: Results of the best fitted clines (simple cline model in all cases) in the two studied transects. Distance between snails over a one-dimensional path was inferred following the procedure developed in Westram et al. (2021). The center and the width of the clines are given in meter. The phenotypic clines were fitted using the measurement of the largest diameter from individual shell picture in ImageJ, and provides estimate of the snail size (in mm) at each end (Dwarf and Large) of the two studied transect. The WGS clines were fitted using the arrangement karyotype (Arrangement) and *Ak* genotype (Ak^{120}, Ak^{100}, Ak^{80}) of each snail obtained from the clustering analyses (see Method). The allozyme clines were fitted using the allozyme allele frequencies found in 7 sites sampled along the southern transect by Tatarenkov and Johannesson (1999), which were placed on our one dimensional path (see method). The WGS and allozyme clines provide estimates of arrangement or allele frequencies at each end (Dwarf and Large) of the two studied transect and also included a fit where the Ak^{80} and Ak^{100} alleles located on the same arrangement were pooled (Ak^{80+100}).

Transect	Data type	Fitted data	Centre	Width	Dwarf	Large
South	Phenotype	Shell size	105.86 [92.75 -125.64]	41.97 [9.82-134.14]	8.85 [8.52-9.17]	10.98 [10.44-11.89]
	WGS	Arrangement	116.49 [110.00-123.20]	76.78 [51.77-100.83]	1.00 [1.00-1.00]	0.00 [0.00-0.00]
		Ak^{120}	116.49 [110.00-123.20]	76.78 [51.77-100.83]	1.00 [1.00-1.00]	0.00 [0.00-0.00]
		Ak^{100}	129.60 [121.20-139.87]	105.32 [80.47-143.5]	0.00 [0.00-0.00]	1.00 [1.00-1.00]
		Ak^{80}	118.97 [108.54-123.56]	1.35 [0.00-61.76]	0.01 [0.00-0.03]	0.19 [0.12-0.28]
		$Ak^{80/100}$	116.49 [110.00-123.20]	76.78 [51.77-100.83]	0.00 [0.00-0.00]	1.00 [1.00-1.00]
	Allozyme	Ak^{120}	65.79 [57.6-76.19]	95.97 [56.34-124.99]	0.99 [0.98-1.00]	0.05 [0.00-0.11]
		Ak^{100}	74.07 [62.2-84.62]	73.48 [25.63-123.09]	0.05 [0.00-0.14]	0.75 [0.68-0.85]
		Ak^{80}	42.18 [20.37-95.82]	72.97 [0.00-242.14]	0.00 [0.00-0.03]	0.19 [0.14-0.29]
		Ak^{80+100}	70.13 [61.14-79.32]	88.41 [49.12-123.21]	0.03 [0.00-0.12]	0.95 [0.88-1.00]
North	Phenotype	Shell size	64.50 [47.36-74.98]	51.37 [15.47-111.11]	8.24 [7.05-8.89]	11.48 [11.16-11.90]
	WGS	Arrangement	66.46 [60.46-72.48]	52.93 [38.35-74.78]	1.00 [1.00-1.00]	0.09 [0.04-0.16]
		Ak^{120}	66.46 [60.46-72.48]	52.93 [38.35-74.78]	1.00 [1.00-1.00]	0.09 [0.04-0.16]
		Ak^{100}	66.69 [58.72-74.93]	55.59 [37.78-83.7]	0.00 [0.00-0.00]	0.77 [0.69-0.85]
		Ak^{80}	64.10 [45.75-81.50]	1.35 [0.00-139.00]	0.01 [0.00-0.04]	0.13 [0.09-0.17]
		$Ak^{80\ 100}$	66.46 [60.46-72.48]	52.93 [38.35-74.78]	0.00 [0.00-0.00]	0.91 [0.84-0.96]

Table S5: Variation of frequency and estimates of F_{IS} of the karyotype inferred from the clustering analyses (Figure 2c over) using seven bins at equal distance along the northern and southern transects. In order of appearance, the transect, the start and the end of the bin, the number of snails in the bin, the observed frequency of heterozygotes, the expected frequency of heterozygotes if the snails inside the bin were at HWE, the expected frequency of heterozygotes from the cline fit, the p-value of the F_{IS} from the Chi-square test using the cline expectation, the F_{IS} calculated from the cline expectation, the p-value of the F_{IS} from the Chi-square test using the HWE expectation from the snails inside the bin, and the F_{IS} from HWE expectation inside the bin. Bold line highlights bins containing the centre of the allelic cline in each transect.

Site	N	start	end	Obs_het	Exp_HWE	Exp_cline	P-value_cline	Fis_cline	P-value_HWE	Fis_HWE
North	17	0,00	25,80	0,00	0,00	0,04	1,00	NA	NA	NA
	22	25,80	51,60	0,09	0,30	0,22	0,46	0,59	0,14	0,69
	**36**	**51,60**	**77,40**	**0,33**	**0,49**	**0,46**	**0,56**	**0,28**	**0,41**	**0,31**
	36	77,40	103,19	0,22	0,35	0,36	0,38	0,38	0,38	0,36
	20	103,19	128,99	0,30	0,26	0,20	0,72	-0,50	0,77	-0,18
	16	128,99	154,79	0,19	0,17	0,17	1,00	-0,08	0,93	-0,10
	19	154,79	180,59	0,16	0,15	0,17	1,00	0,06	0,94	-0,09
South	13	0,00	24,20	0,00	0,00	0,01	0,94	0,00	NA	NA
	16	24,20	48,41	0,00	0,00	0,03	0,77	0,00	NA	NA
	12	48,41	72,61	0,08	0,22	0,11	0,63	0,27	0,52	0,62
	16	72,61	96,81	0,13	0,22	0,28	0,53	0,56	0,64	0,43
	**28**	**96,81**	**121,01**	**0,14**	**0,48**	**0,47**	**0,03**	**0,69**	**0,02**	**0,70**
	28	121,01	145,22	0,39	0,42	0,42	0,98	0,05	0,97	0,07
	14	145,22	169,42	0,14	0,13	0,18	0,88	0,22	0,96	-0,08

Table S6: Distribution of arginine kinase (AK) allozyme allele frequencies in samples from France, Wales and Sweden earlier published in Tatarenkov & Johannesson 1994 and Tatarenkov & Johannesson 1999.

		**AK allele frequencies**						
**Area**	**Sample**	**130**	**120**	**110**	**100**	**80**	**N**	**Ref.**
Roscoff (France)	Moderately exposed sites				1.000		63	*a)*
Great Castle Bay, Dyfed (Wales)	Moderately exposed site				1.000		24	*a)*
Saltö (Sweden)	Moderately exposed site		0.096		0.750	0.154	20	*b)*
Lökholmen (Sweden)	Moderately exposed site		0.059		0.735	0.206	34	*b)*
Jutholmen (Sweden)	Moderately exposed site		0.071	0.018	0.750	0.161	28	*b)*
Burholmen (Sweden)	Moderately exposed site				0.850	0.150	20	*b)*
Ursholmen (Sweden)	Moderately exposed site		0.183	0.017	0.650	0.150	30	*b)*
Svangen (Sweden)	Moderately exposed site				0.942	0.038	26*	*b)*
Grundskär (Sweden)	Moderately exposed site				0.864	0.136	33	*b)*
Roscoff (France)	Sheltered sites	0.028	0.958	0.014			36	*a)*
Gann Flats, Dyfed (Wales)	Sheltered site	0.018	0.982				28	*a)*
Långholmen (Sweden)	Sheltered site		0.809	0.059	0.103	0.029	34	*b)*
Lökholmen (Sweden)	Sheltered site	0.034	0.724	0.103	0.121	0.017	29	*b)*
Jutholmen (Sweden)	Sheltered site	0.022	0.870	0.087	0.022		23	*b)*
Burholmen (Sweden)	Sheltered site		0.694		0.250	0.056	18	*b)*
Ursholmen (Sweden)	Sheltered site		0.861	0.083	0.056		18	*b)*
Svangen (Sweden)	Sheltered site		0.620	0.060	0.320		25	*b)*
Grundskär (Sweden)	Sheltered site		0.827		0.173		26	*b)*

*A rare 90 allele present at frequency of 0.019

a) Data originally presented in Tatarenkov & Johannesson 1999 but in this publication alleles 80 and 100 were pooled into an "exposed" allele. We have now extracted the allele frequency information from the unpublished genotype information of the original study, and confirmed that the 80 allele of AK was not found in the samples from Roscoff and Wales.

b) Data from Tatarenkov & Johannesson 1994. The Swedish sites are small islands on the Swedish west coast within 15 km distance where were sampled for a comparison between sheltered and moderately exposed environments. The data shown here is from a subsample of an extensive set of local samples from this area. All data is presented in a published appendix of the original paper.

References:

Tatarenkov A. & Johannesson K. 1994. Habitat related allozyme variation on a microgeographical scale in the marine snail *Littorina mariae* (Prosobranchia: Littorinacea). Biol. J. Linn. Soc. 53:105-125

Tatarenkov A. & Johannesson K. 1999. Micro- and macrogeographical allozyme variation in *Littorina fabalis*; do sheltered and exposed forms hybridize? Biol. J. Linn. Soc. 67: 199-212.

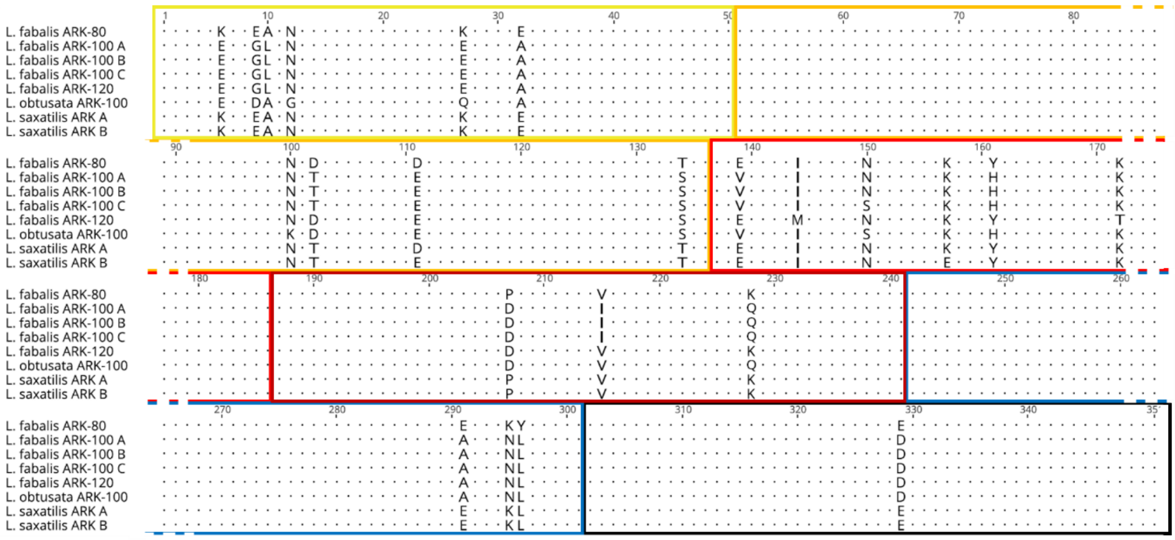

Figure S1: Illustration of amino acid replacements in the protein sequences of AK allozymes in *L. fabalis*, *L. obtusata* and *L. saxatilis* characterized from the cDNA sequencing. Exons are delimitated by the colored rectangles (yellow = exon 1, orange = exon 2, red = exon 3, brown = exon 4, darkblue = exon 5, black = exon 6).

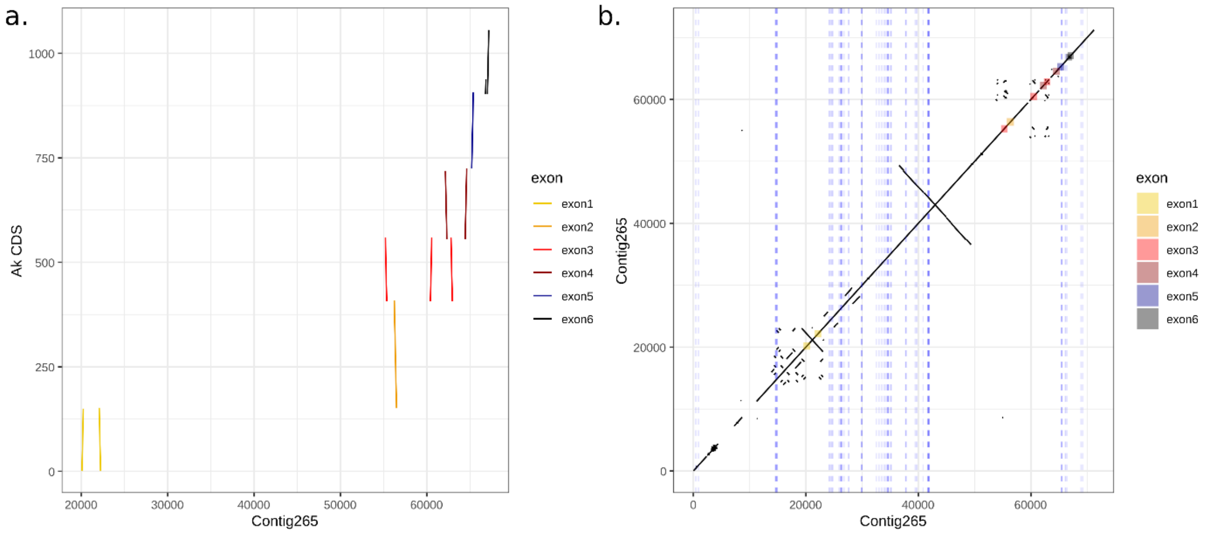

Figure S2: Dot plots to check quality of the assembly of contig265 of the *L. saxatilis* reference genome and positions of the *Ak* exons on this contig. a. The *Ak* exon sequences, from cDNA, aligned against the contig265 sequence, showing that multiple exons (exon 1, exon 3, exon 4 and part of exon 5) are duplicated in the current assembly. b. Contig265 aligned against itself. The colored rectangles center around the *Ak* exons, but are made 1kb larger than the exon size for ease of visualization. The black lines show that some homologous sequences have likely been assembled as inverted repeats in the reference genome ("cross" patterns), one of which includes the *Ak* exon 1 (in yellow) from 20kb to 22kb. Other regions show tandem repeats (parallel lines). Gaps represent runs of Ns. The horizontal dashed blue lines show the positions of the 70 SNPs found on contig265 from the WGS.

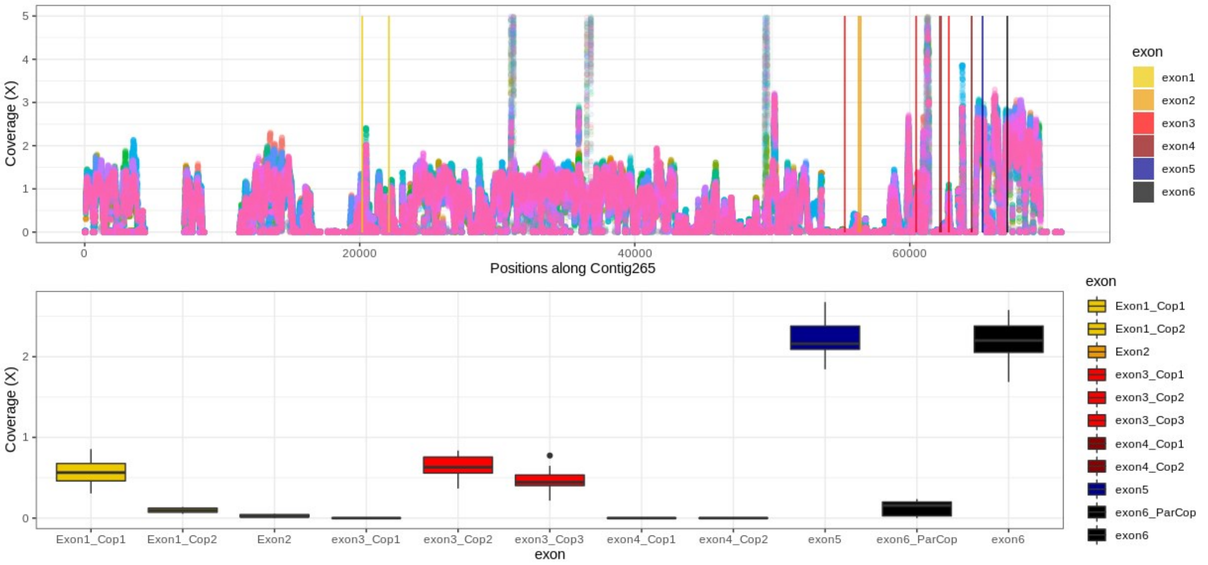

Figure S3: Top: variation in relative coverage (X) of each base along the contig265 for the 12 most covered individuals (one color per individuals), with the positions of the *Ak* exons highlighted by the vertical colored lines (see color legend) and bottom: boxplot of the mean individual coverage for each exon copy or partial copy. The relative coverage was calculated for each individual by dividing the sequenced coverage of each base by the mean coverage of the individual over the 58,246 linked-SNPs that were kept after the filtration procedure.

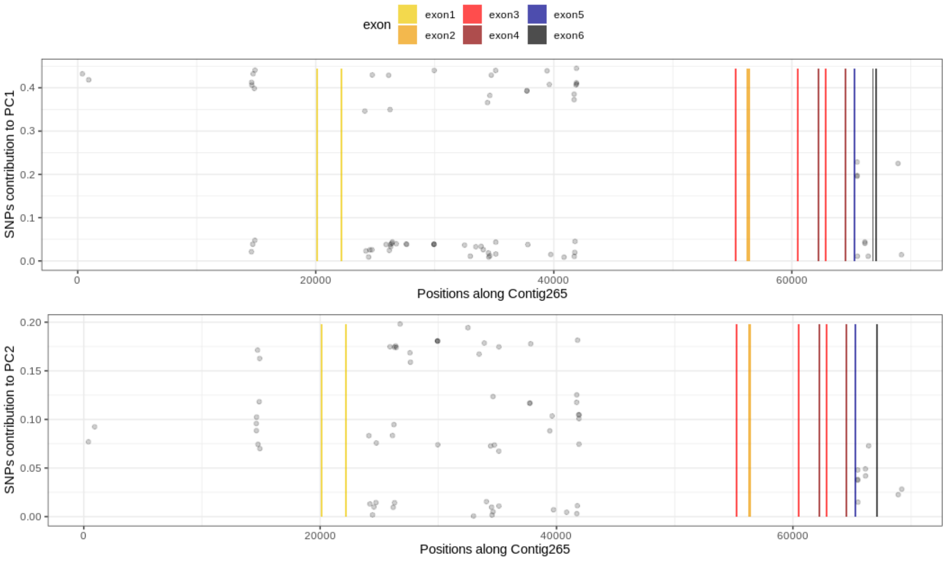

Figure S4: Location and contribution of the 70 SNPs (loading values) from contig265 to the dispersion of the samples over the PC1 and PC2 in Figure 1b. The colored lines show the positions of the *Ak* exons.

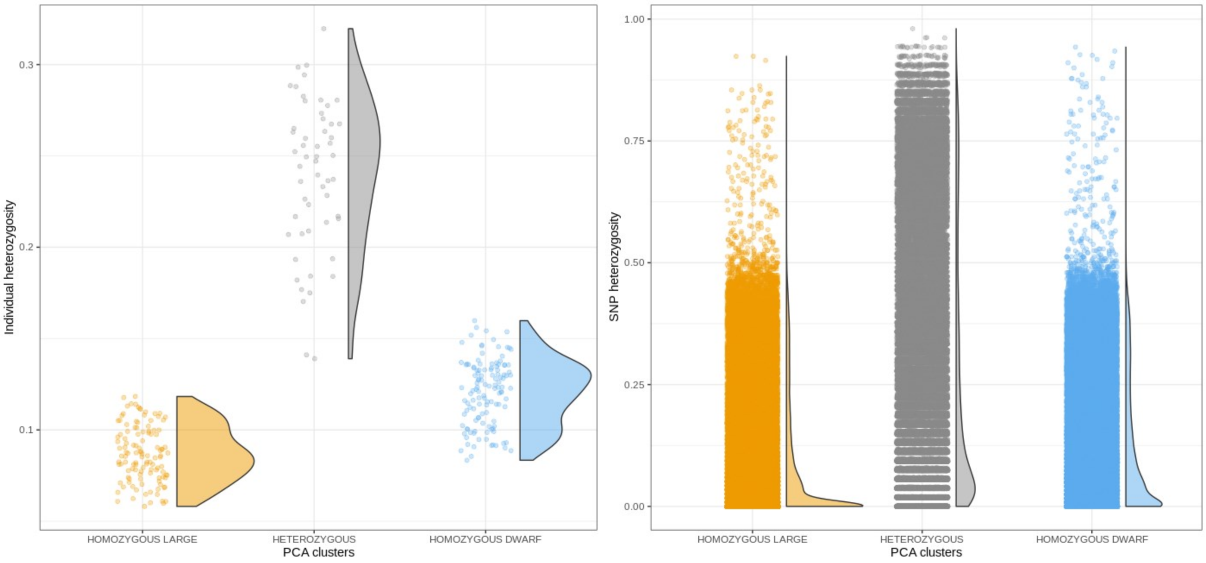

Figure S5: The observed heterozygosity for the 295 snails (left) and for the 58,258 SNPs on LG3 (right), without pruning for close linkage. The observed heterozygosities were computed independently for each PCA cluster described in Figure 2c. The yellow plot is mostly composed of large individuals, homokaryotypes for the arrangement found in the large ecotype (L/L). The blue plot is mostly composed of dwarf individuals, homokaryotypes for the other arrangement (D/D), and the grey plot is composed of individuals that are putatively heterokaryotypes (L/D) for the inversion. Differences of individual heterozygosity between the homokaryotype L/L and D/D (left) are highly significant (t test: t = -15.673, df = 235.51, p-value < 2.2e-16).

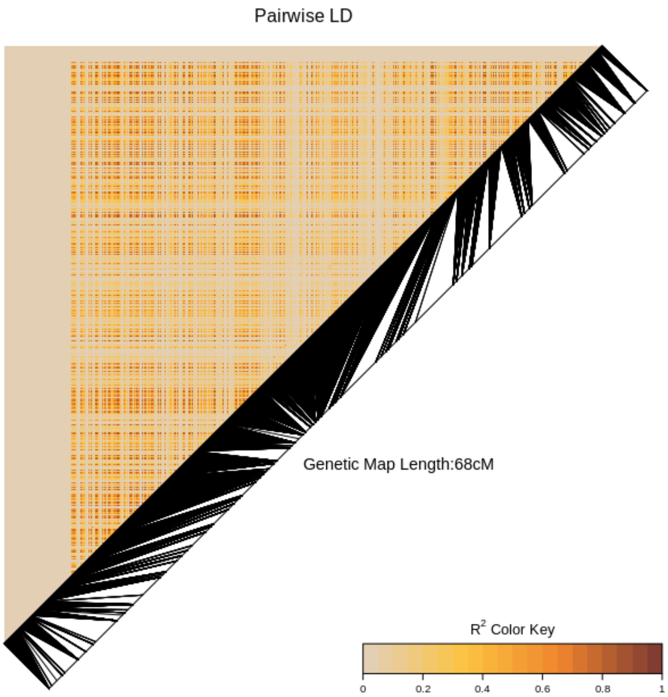

Figure S6: Pairwise Linkage Disequilibrium over the inversion between 7148 SNPs filtered for a maf of 0.1 and minimum 1kb distance. The color gradient of the heatmap represents the LD value estimated from allelic correlation, R^2 (brown=1, beige=0). Each SNPs are positioned on the black scale according to their contig position on the *L. saxatilis* linkage map.

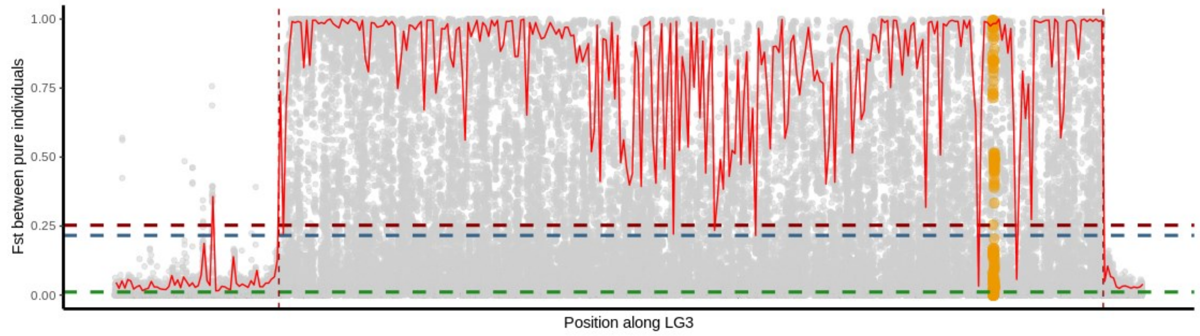

Figure S7: F_{ST} value along LG3 between the most distant PCA clusters in Figure 2c. Each dot corresponds to the F_{ST} value estimated for one SNP. SNPs from the contig containing *Ak* are highlighted in orange. The red line shows the variation of the upper F_{ST} 5% quantile over bins of 10kb. The horizontal lines in blue, green and dark red show the average F_{ST} values over the entire contig, outside and inside the main island of differentiation, respectively. The limits of the island were defined by eye and are shown on the graph by the two vertical dashed lines.

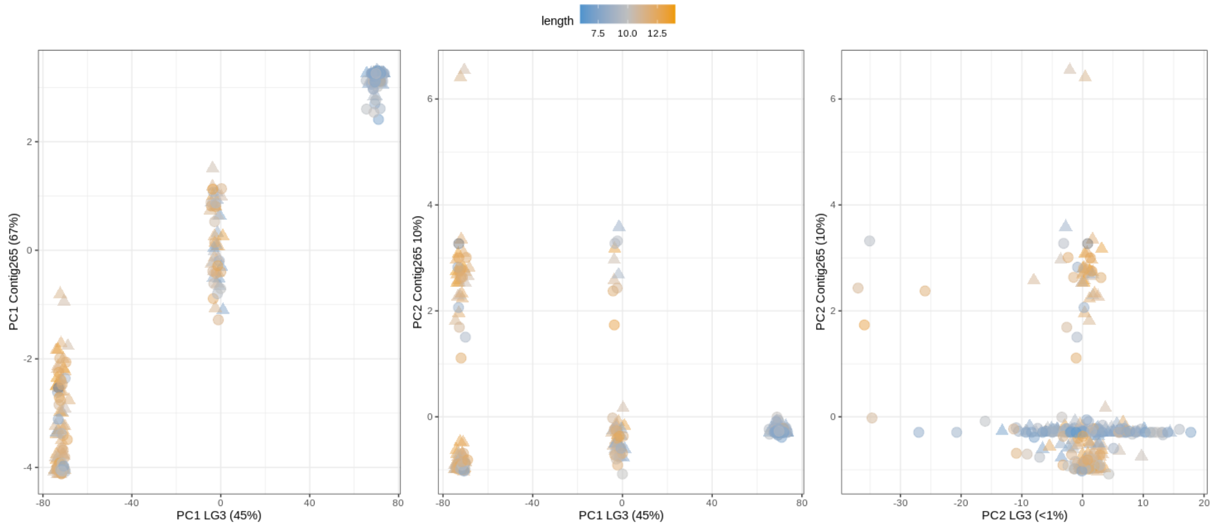

Figure S8: Correlation between the individual snail positions in first and second principal components (PC1 and PC2) performed on the 70 SNPs from contig265 and on the 9,905 SNPs from the whole LG3. Snails are colored by their size. The snail positions of PC1 and PC2 from the contig265 correlated with the PC1 of the whole LG3, but not PC2.

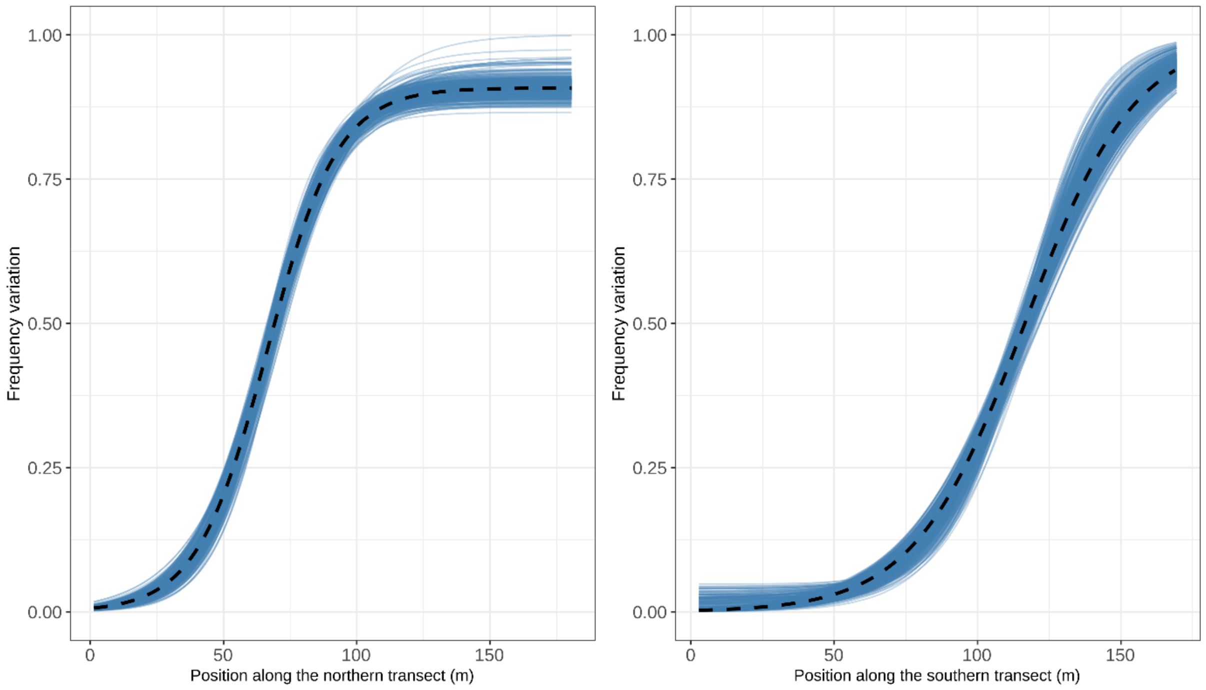

Figure S9: Results of the cline fit for the 484 arrangement diagnostic SNPs (with an F_{ST} value of 1 between homokaryotype individuals) for the northern transect (left) and the southern transect (right). The back dashed line represent the inversion frequency cline shown also in figure 4c,d.

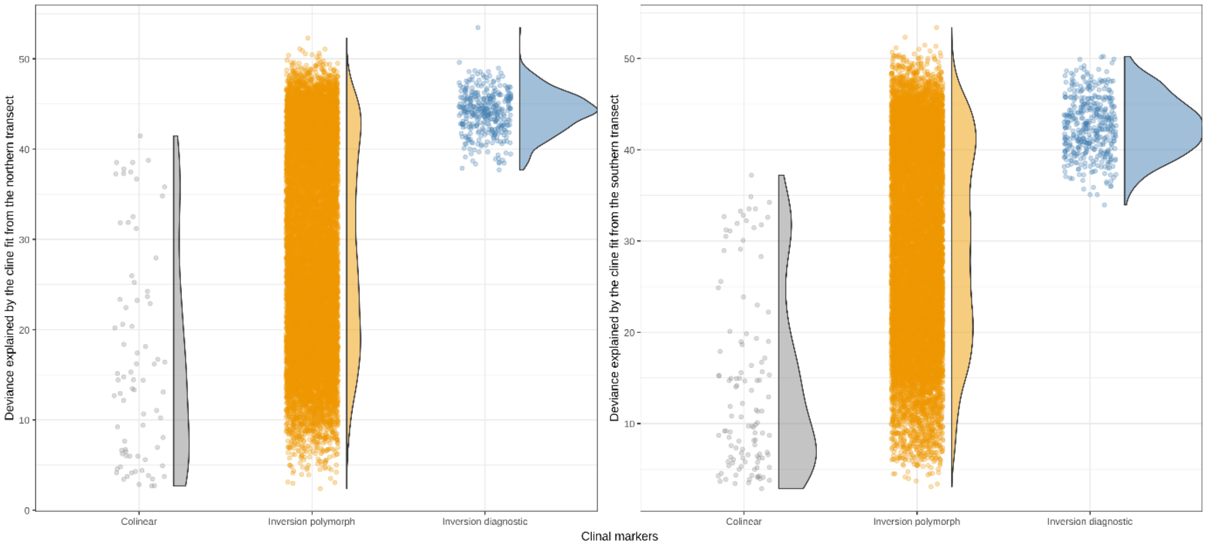

Figure S10: Goodness of fit for the individuals SNP cline fitting clines, expressed as the proportion of the deviance explained by the cline using a generalize linear regression model (GLM) with binomial error for the northern transect (left) and the southern transect (right). Each dot corresponds to the deviance explained by the cline fit at one given SNP. SNPs located outside the inversion are colored in grey, and SNPs inside the inversion are colored in orange or blues depending if they are differentially fixed between arrangement (blue) or polymorph (orange).

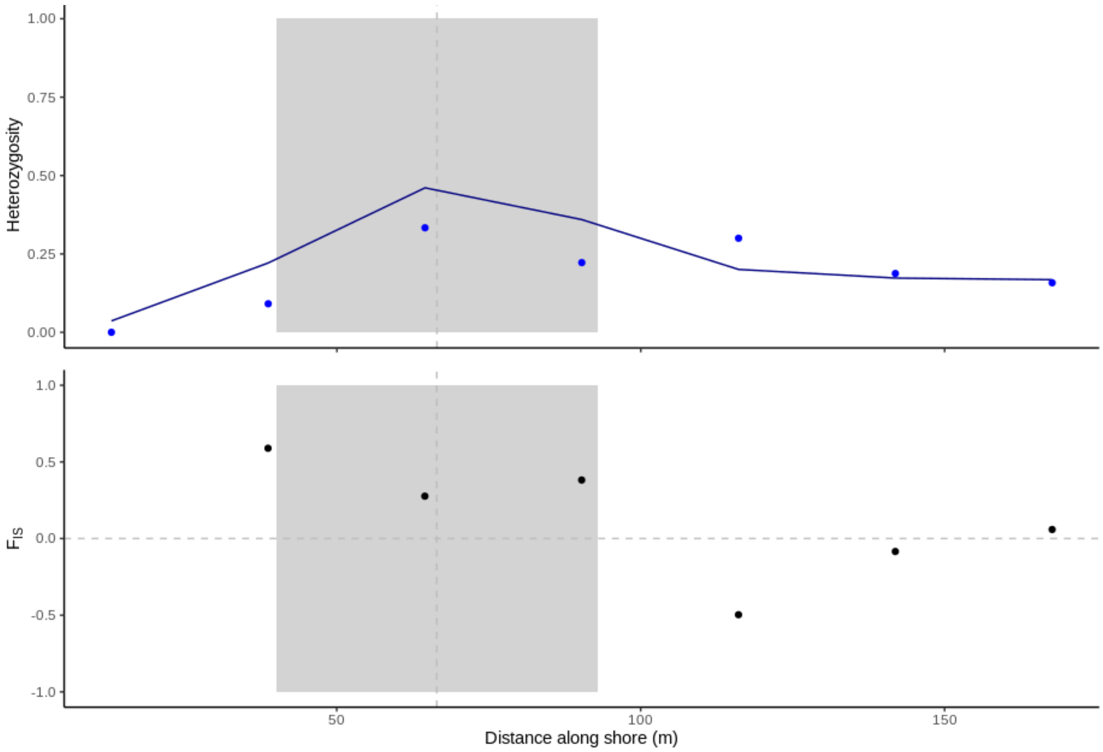

Figure S11: Top: Variation of heterozygosity observed (blue dots) and expected from the cline fits (blue line) over 7 bins of equal distances along the northern transect. Bottom: Variation in F_{IS} over the 7 bins along the northern transect. The grey boxes show the limits of the cline width.

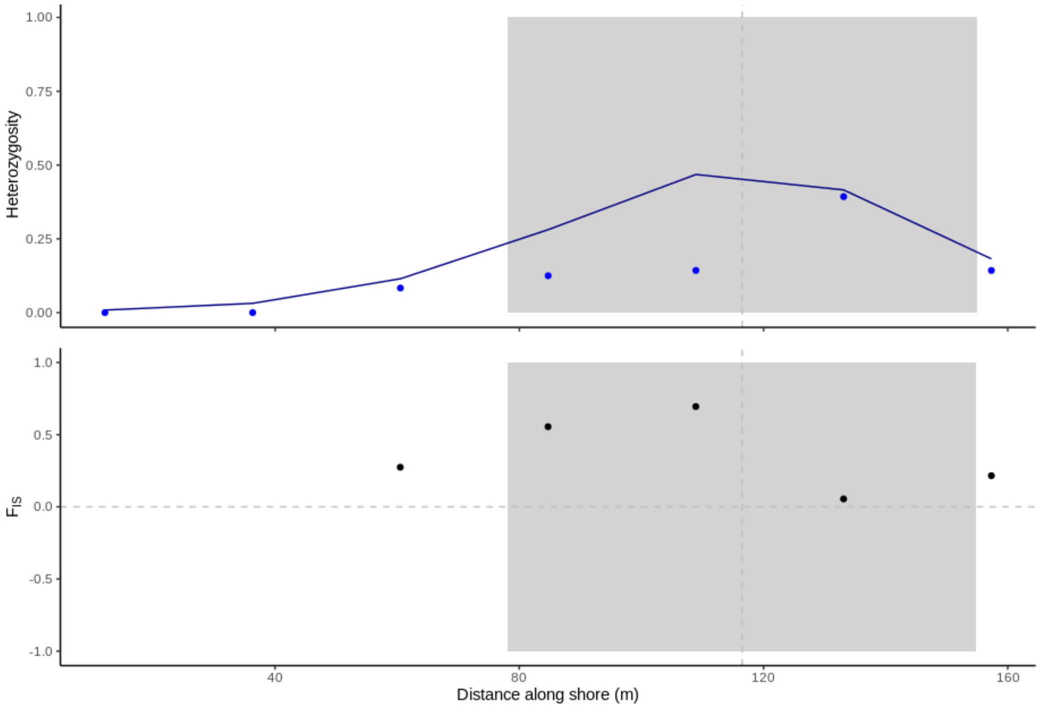

Figure S12: Top: Variation in heterozygosity observed (blue dots) and expected from the cline fits (blue line) over 7 bins of equal distances along the southern transect. Bottom: Variation in F_{IS} over the 7 bins along the southern transect. The grey boxes show the limits of the cline width.
